# Nanoparticles targeting hematopoietic stem and progenitor cells: Multimodal carriers for the treatment of hematological diseases

**DOI:** 10.3389/fgeed.2022.1030285

**Published:** 2022-11-02

**Authors:** Luis J. Cruz, Somayeh Rezaei, Frank Grosveld, Sjaak Philipsen, Christina Eich

**Affiliations:** ^1^ Translational Nanobiomaterials and Imaging, Department of Radiology, Leiden University Medical Center, Leiden, Netherlands; ^2^ Erasmus University Medical Center, Department of Cell Biology, Rotterdam, Netherlands

**Keywords:** hematopoietic stem cells, nanoparticles, targeting, gene therapy, imaging, delivery

## Abstract

Modern-day hematopoietic stem cell (HSC) therapies, such as gene therapy, modify autologous HSCs prior to re-infusion into myelo-conditioned patients and hold great promise for treatment of hematological disorders. While this approach has been successful in numerous clinical trials, it relies on transplantation of *ex vivo* modified patient HSCs, which presents several limitations. It is a costly and time-consuming procedure, which includes only few patients so far, and *ex vivo* culturing negatively impacts on the viability and stem cell-properties of HSCs. If viral vectors are used, this carries the additional risk of insertional mutagenesis. A therapy delivered to HSCs *in vivo*, with minimal disturbance of the HSC niche, could offer great opportunities for novel treatments that aim to reverse disease symptoms for hematopoietic disorders and could bring safe, effective and affordable genetic therapies to all parts of the world. However, substantial unmet needs exist with respect to the *in vivo* delivery of therapeutics to HSCs. In the last decade, in particular with the development of gene editing technologies such as CRISPR/Cas9, nanoparticles (NPs) have become an emerging platform to facilitate the manipulation of cells and organs. By employing surface modification strategies, different types of NPs can be designed to target specific tissues and cell types *in vivo*. HSCs are particularly difficult to target due to the lack of unique cell surface markers that can be utilized for cell-specific delivery of therapeutics, and their shielded localization in the bone marrow (BM). Recent advances in NP technology and genetic engineering have resulted in the development of advanced nanocarriers that can deliver therapeutics and imaging agents to hematopoietic stem- and progenitor cells (HSPCs) in the BM niche. In this review we provide a comprehensive overview of NP-based approaches targeting HSPCs to control and monitor HSPC activity *in vitro* and *in vivo*, and we discuss the potential of NPs for the treatment of malignant and non-malignant hematological disorders, with a specific focus on the delivery of gene editing tools.

## 1 Introduction

### 1.1 Scope of this review

**FIGURE 1:**
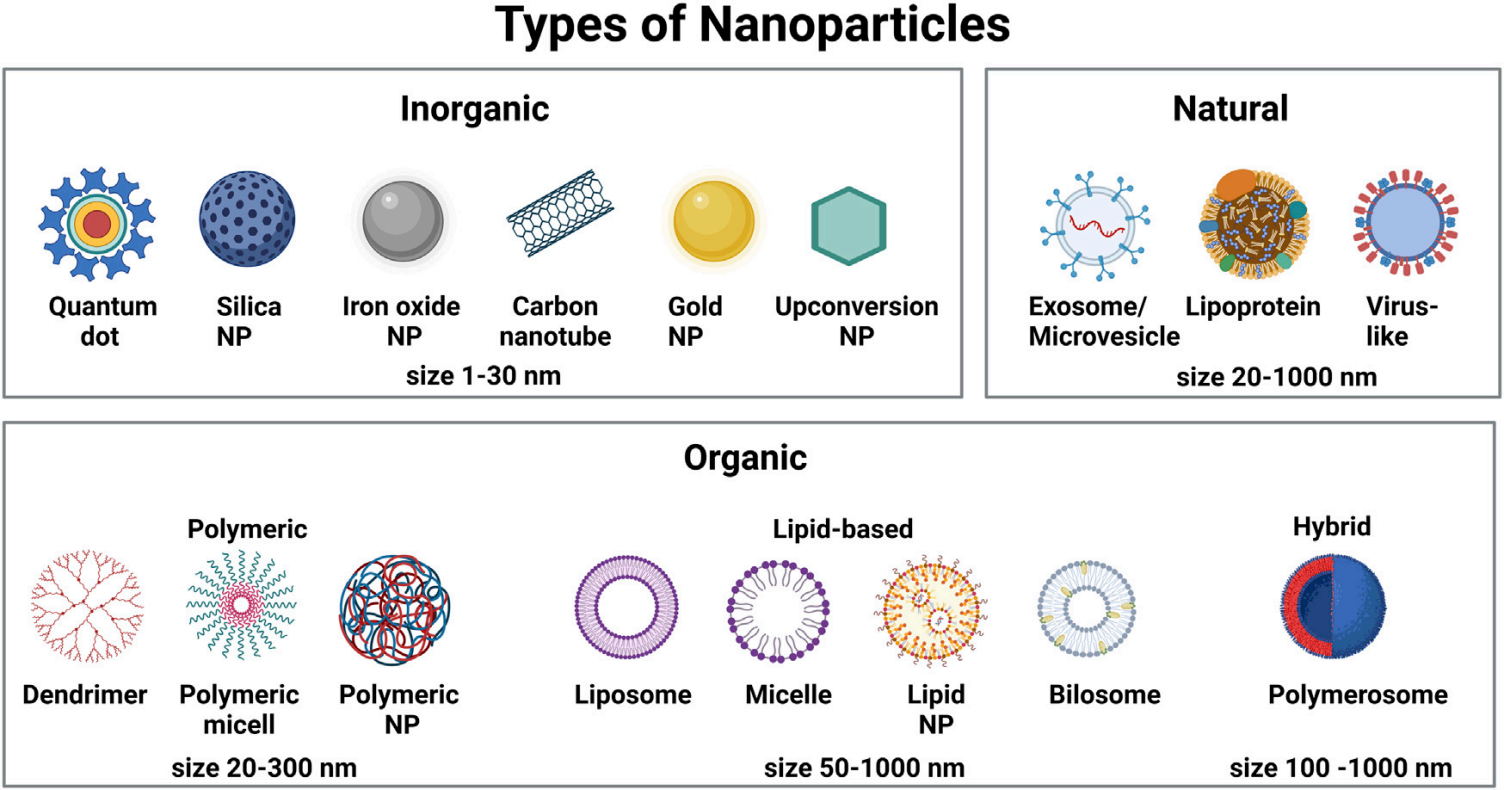
Schematic representation of different types of nanoparticles commonly used to deliver therapeutics and imaging agents in biomedical applications.

Hematopoietic stem cells (HSCs) have the capacity to replenish all blood cell lineages during the steady-state cellular turnover of the blood system, and under stress conditions such as acute inflammation or HSC mobilization (Orkin and Zon, 2008; Sun et al., 2014; Rodriguez-Fraticelli et al., 2018). Distinct lineage-committed hematopoietic progenitor cells (HPCs) emerge from an individual HSC through many differentiation steps and cell divisions, while HSCs are also maintained through self-renewal (Orford and Scadden, 2008; Notta et al., 2016; Velten et al., 2017; Rodriguez-Fraticelli et al., 2018). Hereditary hematological disorders (including hemophilia, blood clotting disorders, thalassemias and sickle cell disease) and acquired disorders (including myelodysplastic syndromes and malignancies such as lymphomas, leukemias and myelomas) are examples of pathologies affecting the hematopoietic system, many of which are caused by mutations (Bao et al., 2019). One way to treat these disorders is replacement of diseased HSCs by healthy allogeneic HSCs (Gyurkocza et al., 2010). HSC transplantation is one of the major medical discoveries of the 20th century and has been used for over 50 years for the treatment of leukemias and monogenic blood-related diseases (Copelan, 2006; Appelbaum, 2007). While HSC transplantations save tens of thousands of lives per year worldwide (Gratwohl et al., 2010), many patients remain deprived of this life-saving procedure due to the lack of a compatible donor or an insufficient number of HSCs in the graft. This can lead to treatment-related morbidity and mortality (Mimeault et al., 2007). Alternatively, repair of disease-causing genes in autologous HSCs *ex vivo* followed by HSC transplantation or direct treatment *in vivo* might be the “holy grail” for malignant and non-malignant hematological diseases, provided that sufficient numbers of autologous HSCs could be corrected. In the last decade, in particular with the development of gene editing technologies such as CRISPR/Cas9, nanoparticles (NPs) have become an emerging platform to facilitate the genetic manipulation of cells and tissues *in vitro* and *in vivo*. NPs are submicron-sized particles that can be generated from a variety of components, including polymers, lipids, metals and rare earth elements, or can be isolated as extracellular vesicles (EVs) from cells or assembled from virus capsids (Figure 1). NPs represent promising tools for monitoring and controlling HSC activity *in vivo* due to their capacity to protect a payload from premature degradation and mediate endosomal escape to enable nucleic acid and Cas9 translocation to the cytoplasm and nucleus (Yin et al., 2014), while circumventing efficacy and safety issues of classical viral vehicles. Moreover, recent developments in nanotechnology have demonstrated the feasibility of site-specific delivery by smart polymers featuring spatiotemporal release kinetics (Zhuo et al., 2021), which could facilitate the manipulation of HSCs *in situ* while limiting off-target delivery.

Several NP formulations have been approved for clinical use, mostly as a delivery vehicle for therapeutics in the field of cancer and regenerative medicine, as a delivery platform for medical imaging agents, or as a vaccine in the field of infectious diseases (Anselmo and Mitragotri, 2019). NP-based diagnostics and therapies have also received considerable attention in the field of hematological disorders, such as for the detection of circulating tumor cells by anti-CD20-coated quantum dots (Shariatifar et al., 2019), for the treatment of anemia by orally administered iron-based NPs or the manipulation of hematopoietic stem- and progenitor cells (HSPCs) in the fetal and adult hematopoietic niche (Zariwala et al., 2013; Hosny et al., 2015).

In this review, we provide a comprehensive overview of NP-based approaches targeting HSPCs in biomedical applications. In the first part, we focus on how HSPCs interact with NPs, taking into account the specific biology of HSCs and HPCs, and their localization in hematopoietic niches. In the second part, we review the use of NPs to control and monitor HSPC activity *in vitro* and *in vivo*. In the third part of this review, we discuss the potential of NPs for the treatment of malignant and non-malignant hematological disorders, with a specific focus on the delivery of gene editing tools.

### 1.2 Nanoparticles

NPs are widely accepted to have a size between 5–300 nm (although structures of up to 1,000 nm have also been reported). Based on their chemical composition, they are commonly grouped into carbon-based NPs (carbon nanotubes and fullerenes), inorganic NPs (quantum dots, metallic NPs, rare earth-material NPs) and organic NPs (lipid NPs, polymeric NPs and EVs) (Figure 1). The use of NPs as drug delivery system has many advantages over the delivery of naked drugs: 1) due to their large inner volume, NPs can be loaded with hydrophilic and hydrophobic compounds, including fluorophores, metals, peptides, proteins, nucleic acids or biomimetic molecules, which can increase the concentration of these compounds locally; 2) drug encapsulation in NPs can improve the biocompatibility and stability of conventional drugs and overcome problems of insolubility; 3) in contrast to conventional drugs, NPs present an enhanced circulation time in the blood stream; 4) NPs can be designed to be multifunctional by exerting both diagnostic and therapeutic actions; and 5) the NP surface can be functionalized with targeting moieties to permit site- and/or cell-specific payload delivery and improve the ratio of efficacy/cytotoxicity of the encapsulated payload. This reduces adverse side effects often associated with systemically applied high doses of drugs.

### 1.3 Nanoparticle uptake

NP uptake is influenced by three main factors: 1) The physicochemical properties of NPs, such as size, polydispersity index (measure of the heterogeneity of a sample based on size), shape, charge, surface modification and surface hydrophobicity/hydrophilicity; 2) The physiological properties of target cells and their microenvironment (e.g., presence of cell surface proteoglycans or receptors, levels of serum proteins); and 3) experimental factors, including temperature, incubation time, osmolarity and ionic strength (He et al., 2021).

**FIGURE 2:**
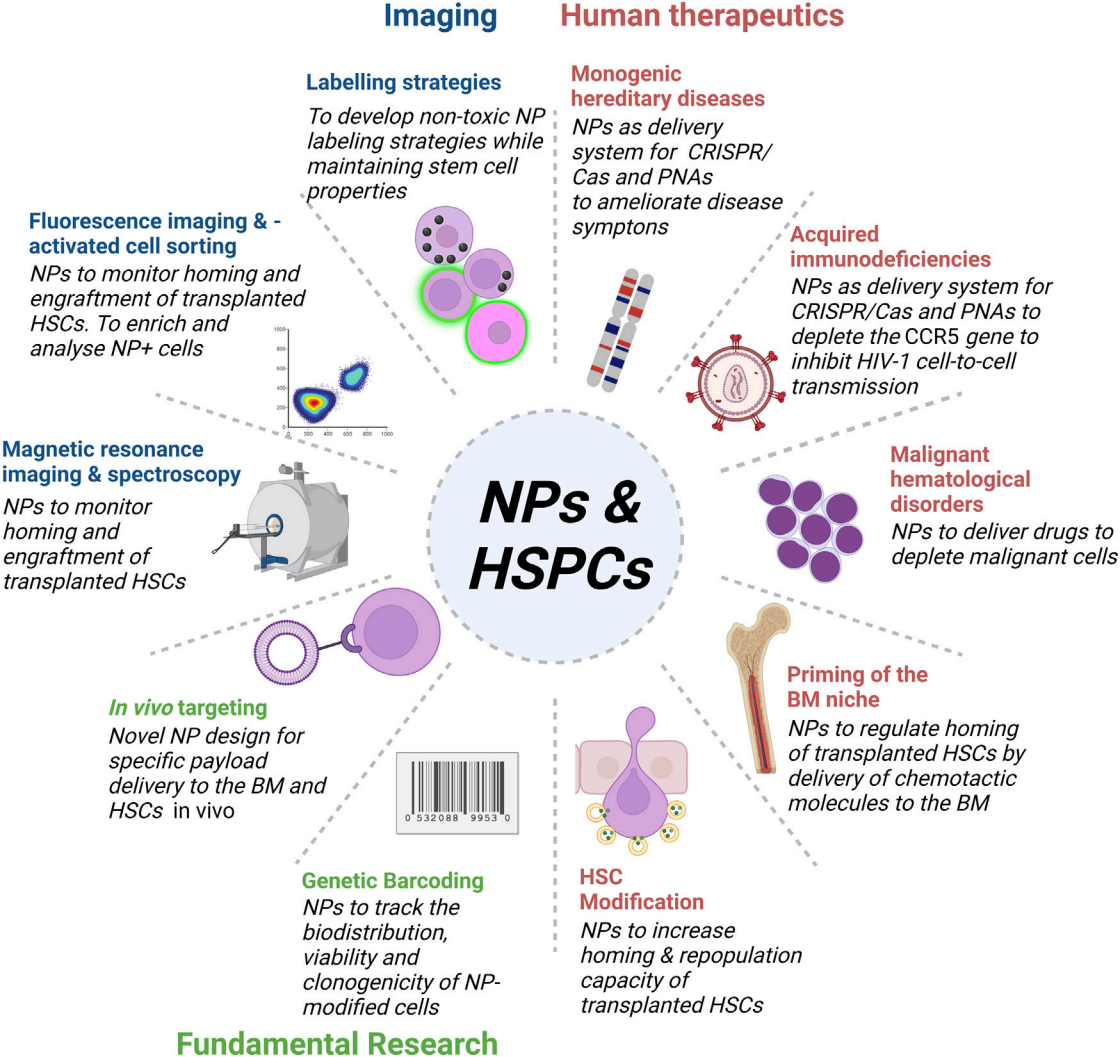
Overview of NPs targeting HSPCs in bioimaging, fundamental research and the development of human therapeutics. Development of novel NP designs and bioconjugation strategies enables the application of multifunctional NPs for in *in vivo* imaging and therapy of HSPCs.

After encounter with the cell membrane, NPs are taken up *via* the cellular endocytosis machinery by two main mechanisms, phagocytosis and pinocytosis. Phagocytosis is the preferred uptake mechanism for larger particles (>500 nm), such as pathogens, cell fragments, or NPs. Pinocytosis (including macropinocytosis, clathrin-mediated endocytosis, caveolae-mediated endocytosis and clathrin- and caveolin-independent endocytosis) is regarded as the dominant mechanism for the uptake of NPs of less than 500 nm (Zhao and Stenzel, 2018). Positively charged NPs are internalized rapidly *via* the clathrin-mediated pathway, while negatively charged NPs are internalized mainly through pathways other than clathrin and caveolin (Harush-Frenkel et al., 2007). However, positively charged NPs are in general associated with higher cytotoxicity (Goodman et al., 2004; Oh et al., 2010), thus fast uptake rates are not necessarily beneficial. Current NP-based approaches targeting HSCs in biomedical applications focus around three major objectives (Figure 2). Firstly, improving labelling strategies to monitor transplanted HSCs by different imaging modalities. Secondly, the delivery of drugs and gene editing tools to modulate HSCs and the BM niche for the development of human therapeutics, and thirdly, fundamental research to develop novel tools to target and track NPs biodistribution *in vivo*. Examples of NPs that have been used to target HSPCs for therapy or monitoring purposes mainly belong to the group of organic and inorganic NPs (Table 1).

**TABLE 1:** Summary of NP systems targeting HSPCs and the BM *in vitro* and *in vivo*.

Category	NPs	Size (nm)	Target	Purpose	Ref
**Polymeric NPs**	Polystyrene and PLLA NPs	116–131	Human HSPCs	Study the influence of NPs on cell differentiation capacity and functionality	Brüstle et al. (2015)
	Carboxylated polystyrene NPs	40	Human HSPCs	Study NP loading behavior	Deville et al. (2017)
	Chitosan NPs	200–700	Murine BM-derived HSPCs	Study the influence of NP size on cell viability and functionality	Zaki et al. (2015)
	Chitosan NPs	200	Human peripheral blood-derived HSPCs	Study how deacetylation degree and molecular weight of chitosan affect cell viability and functionality	Jesus et al. (2020)
	PLGA-NPs encapsulating fluorine 19 (^19^F)	290	Human cord blood -derived HSPCs	Cell labeling and MR imaging	Duinhouwer et al. (2015)
	Protamine sulfate-modified PLGA-NPs encapsulating perfluoro-1,5-crown ether	210	Human HSPCs	Cell labeling and MR imaging	Aday et al. (2014)
	PLGA NPs encapsulating Wnt3a protein	178	ESCs	Delivery and stabilization of Wnt3a protein	Tuysuz et al. (2017)
	Chitosan/tripolyphosphate/fucoidan NPs encapsulating SDF-1	173–403	BM-MSCs	Delivery of SDF-1	Huang and Liu, (2012)
	Alendronate-modified PLGA-PEG-NPs encapsulating bortezomib	150–200	Bone	Drug delivery to BM	Swami et al. (2014)
	Triblock co-polymer Poloxamer-40-modified polystyrene NPs	60, 150, 250	Rabbit bone marrow (BM)	Targeting the BM	Porter et al. (1992)
	PLGA-NPs encapsulating CRISPR RNPs	300–400	Human HSPCs	Delivery of CRISPR RNPs to edit the β-globin gene locus	Cruz et al. (2021)
	Poly-β-amino ester NPs encapsulating CRISPR RNPs	200	GCSF-mobilized human CD34^+^ HSPCs	Delivery of CRISPR RNPs to edit the CD33 gene locus	El-Kharrag et al. (2022)
	PLGA-NPs encapsulating PNA and DNA NPs	150	HSCs	*In vivo* gene editing of the CCR5 and β-globin gene loci	McNeer et al. (2011); McNeer et al. (2013)
	Membrane glycan-modified carboxylated polystyrene NPs	40, 100, 200	Human HSPCs	Study the influence of NP size and membrane-associated glycans on NP loading behavior	Wathiong et al. (2019)
**Lipid-based NPs**	Liposomes encapsulating USPIO (P7228)	20–50	Human cord blood-derived HSPCs	MR imaging of prelabeled HSPCs in the BM	Daldrup-Link et al. (2005)
	Liposomes encapsulating Wnt3a protein	130–150	ESCs	Delivery and stabilization of Wnt3a protein	Tuysuz et al. (2017)
	Maleimide headgroup-modified liposomes and liposome-like synthetic NPs encapsulating GSK-3β inhibitor	230	Conjugated to murine HSCs	Cell engineering/adjuvant delivery to improve outcome of HSC transplantation	Stephan et al. (2010)
	PEG-lipid NPs encapsulating siRNA (“BM1”)	60–80	Murine BMECs	Gene silencing in BMECs	Sago et al. (2018)
	Lipid–PEG NPs encapsulating small siRNA (NicheEC-15″)	60–80	Murine BMECs	Gene silencing in BMECs	Krohn-Grimberghe et al. (2020)
	Alendronate-modified liposomes encapsulating SDF-1 gene	116, 123	Bone/Osteoblasts	Bone targeted plasmid delivery for ectopic gene expression	Chen et al. (2018)
	Lipid NPs encapsulating Cas9 mRNA and sgRNAs		Human HSCs	Gene editing of BM cells	Intellia Therapeutics, I., 2021
	RGD-PEG- modified liposomes encapsulating siRNA or doxorubicin	100–210	ECs	SiRNA delivery to ECs	Schiffelers et al. (2005)
	Multilamellar lipid vesicles encapsulating GSK-3β inhibitor	496	Conjugated to murine HSCs	Cell engineering/adjuvant delivery to enhance proliferation kinetics of *in utero* transplanted HSCs	Loukogeorgakis et al. (2019)
	PLGA NPs encapsulating γPNAs and donor DNA	200	Murine HSPCs	*In utero* delivery of PNAs and donor DNAs to correct a disease-causing mutation in the β-globin gene	Ricciardi et al. (2018)
	PLGA NPs encapsulating PNAs and donor DNA	156	Human HSPCs	Delivery of PNAs and DNAs to edit the β-globin gene locus	McNeer et al. (2011)
	Lipid NPs encapsulating CRISPR (Cas9 mRNA and sgRNA)	75	Murine liver	Inhibition of antithrombin by gene editing	Han et al. (2022)
**Inorganic NPs**	PEG-modified mesoporous silica NPs	177	Murine ES cell-derived HPCs	Tracking and real-time imaging of ES cell-derived HPCs during the early phases of engraftment	Sweeney et al. (2018)
	Ferumoxtran, magnetic polysaccharide NPs, transferrin, P7228 liposomes, gadopentetate dimeglumine liposomes SPIO and USPIO NPs	20–40, 100–150	Human cord blood-derived HSPCs	Cell labelling and MR imaging	Daldrup-Link et al. (2003)
	SPIO NPs co-administered with protamine sulfate	216–310	Human CD34+HSPCs	Cell labelling and MR imaging	England et al. (2013)
	Ferumoxides–protamine sulfate complexes		Human peripheral blood-derived HSPCs	Cell labelling and MR imaging	Arbab et al. (2005)
	Fe2O3, Fe3O4, Sb2O3, Au, TiO2, Co and Ag NPs	Fe3O4 (20–30), Fe2O3 (55–65), Sb2O3 (41–91), Au (50–100), TiO2 (20–160), Ag (90–210)	Human BM-derived HSPCs	Cell labelling and toxicity study	Bregoli et al. (2009)
	SPIO ferumoxides NPs	120–180	Human cord blood-derived HSPCs	Cell labelling and MR imaging	Daldrup-Link et al. (2005)
	Fluorophore-conjugated dextran coated iron oxide NPs	80	Human HSPCs	Cell labelling and MR and fluorescent imaging	Maxwell et al. (2008)
	Silica-coated, N-(2- aminoethyl)-3-aminopropyltrimethoxysilane-modified iron NPs	60	Murine HSPCs	Immunomagnetic cell isolation	Liang et al. (2009)
	Fluorescent anti-CD34 antibody-conjugated- Fe3O4/Ag-NPs	30–50	Human BM-derived HSPCs	Cell isolation and detection by electron microscopy	Quynh et al. (2018)
	PEG-modified, guide RNA, Cpf1 (or Cas12) endonuclease, (PEI) and single stranded DNA template-functionalized gold NPs	64	Human HSPCs	Delivery of gene editing components (targeted HDR)	Shahbazi et al. (2019)
**Natural NPs**	Megakaryocytic microparticles carrying plasmid DNA	234, 257	Human HSPCs	Delivery of nucleic acids	Kao and Papoutsakis, (2018)
	Pluronic/platelet microvesicle nanocomplexes stabilized with chitosan-alginate	467	Human HSPCs preloaded with “nanoclouds”	Enhanced homing of transplanted HSCs to the BM	Chander and Gangenahalli, (2020b)
	Baboon envelope pseudotyped “nanoblades” fused to Cas9 RNP complexes	<450	Human CD34^+^ HSPCs	Delivery of the CRISPR RNP complex to edit the WAS gene locus	Gutierrez-Guerrero et al. (2021)
**Hybrid NPs**	Polymer (PGA)-stabilized dCas9-RNP/HDR template NPs	100	Human peripheral blood and induced pluripotent stem cell (iPS)-derived HSPCs	Delivery of gene editing components (targeted HDR)	Nguyen et al. (2020)

### 1.4 Hematopoietic stem cells and hematopoietic progenitor cells

HSCs lack known unique cell surface markers that can be used for straightforward cell isolation. Instead, CD34 is commonly used to study HSPCs in the laboratory and for enrichment prior to BM transplantation (Sutherland et al., 1990; Baum et al., 1992). CD34 is a transmembrane glycoprotein expressed on HSPCs and many vascular endothelial cells (ECs). Thus, in addition to a few HSCs the CD34^+^ fraction includes ECs and immature and mature HPCs that can be further distinguished by additional markers (Doulatov et al., 2012). Progress has been made in characterizing human long-term repopulating HSCs (LT-HSCs) based on engraftment analysis of HSC populations as CD19^−^CD34^+^CD38^−^CD45RA-CD49f+CD90^+^ (Thy-1) (Notta et al., 2016). Index-sorting in combination with RNA sequencing further revealed that especially the CLEC9A^h^iCD34^lo^ subset is enriched in LT-repopulating HSCs (Belluschi et al., 2018). Of note, different markers have been employed in studies in human (CD34^+^CD90^+^CD133+ (Wang et al., 2015; Schiroli et al., 2019; Ferrari et al., 2020) and CD34^+^CD90^+^ (Michallet et al., 2000; Negrin et al., 2000)) *versus* non-human primates (CD34^+^CD90^+^CD45RA (Radtke et al., 2017; Humbert et al., 2019). As most studies on NPs and HSCs were conducted using CD34^+^ HSPCs, caution needs to be taken regarding the interpretation of NP targeting data towards HSCs. In this review, we will refer to CD34^+^ cells as HSPCs, unless stated otherwise.

In contrast to HPCs, HSCs are primarily maintained in a quiescent (G0) state in specialized BM niches (Zhang et al., 2003). This state is accompanied by specific physiological properties, such as cell cycle arrest, reduced transcriptional and translational activity and unique energy metabolism (Passegué et al., 2005; Takubo et al., 2013; Yu et al., 2013; Ito et al., 2016) and distinguishes HSCs from more committed progenitors and mature blood cells (Herbein et al., 1994). HSPCs typically display a high nucleus-to-cytoplasm ratio, with few organelles in the cytoplasm, while more differentiated cells display the opposite (Deliliers et al., 2001). Endosomes are pivotal in the endocytic pathway and an important entry route for NPs into cells (Behzadi et al., 2017; Rees et al., 2019). Thus, the number of endosomes influences the extent of NP uptake and it has been shown that dividing HPCs take up exogenous material much easier than non-dividing quiescent LT-HSCs. Based on their resistance to invasion by certain bacteria human HSCs were long considered as unable to perform macropinocytosis or receptor-mediated phagocytosis and were believed to lack the necessary internalization mechanisms to engulf large amounts of extracellular materials (Kolb-Mäurer et al., 2002). However, viruses, mainly lentivirus (LV, 80–100 nm), adenovirus (AdV, 90–100 nm) and adeno-associated virus (AAV, 25 nm), can transduce human HSCs, as demonstrated by transplantation experiments and in clinical trials (Aiuti et al., 2013; Song et al., 2013; Genovese et al., 2014; Sather et al., 2015; Traxler et al., 2016; Ye et al., 2016; Kanter et al., 2017; Thompson et al., 2018). Endocytosis is the main cellular entry route for viruses lacking a viral envelope (AAV, AdV). Thus, even though HSCs are not outstanding phagocytes, they are equipped to endocytose particles from their surroundings. The size of NPs is similar to that of viruses, and NPs with different physical-chemical properties have been shown to target HSPCs *ex vivo* and *in vivo* (Table 1). The endocytotic activity of HSPCs is also dependent on the source of CD34^+^ cells. Umbilical cord blood progenitor cells showed higher endocytotic and phagocytotic rates compared to BM HSPCs (Lewin et al., 2000).

In order to efficiently deliver imaging reagents or therapeutics to cells, entrapment followed by degradation in acidic compartments of the endo/lysosomal pathway must be prevented. Polymeric NPs, such as those made of poly (lactic-co-glycolic acid) (PLGA), have been shown to escape the endosomal pathway and translocate to the cytosol of human CD34^+^ HSPCs (Cruz et al., 2014; Cruz et al., 2021). Different mechanisms, including membrane fusion, osmotic or mechanic rupture due to NP swelling, and membrane destabilization by pH-responsive NPs have been proposed to underlie endosomal escape and subsequent release of encapsulated payload into the cytosol (Smith et al., 2019). As the endosomal escape of NPs is crucial for the efficacy of cargo delivery, positively charged or pH-sensitive functional groups can be incorporated into NPs to enhance this process (Schmaljohann, 2006; Shinn et al., 2022).

### 1.5 Interaction of nanoparticles with hematopoietic stem and progenitor cells

Several groups investigated whether polymeric NPs are suitable delivery systems for human HSPCs *in vitro* (Table 1). Brüstle et al. studied how different types of polymeric NPs affected the functionality and differentiation capacity of human CD34^+^ HSPCs (Brüstle et al., 2015). Inert polystyrene (without carboxylic groups on the surface) and biodegradable polymeric NPs (PLGA-based) showed high uptake rates in HSPCs without inducing cytotoxicity. The cellular NP content was reduced due to consecutive proliferation events during lineage commitment. The differentiation potential of HSPCs was not affected, however mRNA expression of some lineage markers was altered. The significance of this finding needs further investigation.

Deville et al. investigated the short-term interaction and uptake kinetics of carboxylated polystyrene NPs in CD34^+^ HSPCs (Deville et al., 2017). Interestingly, in contrast to dendritic cells, which showed increased NP uptake over time, NP uptake in HSPCs reached a maximum within 1 h and declined afterwards, suggesting an energy-dependent cellular process that actively controls uptake and release of particles (Deville et al., 2017). NPs made of the natural compound chitosan, the only biopolymer that is positively charged at low pH, have considerable potential as delivery system for HSCs based on their ability to deposit negatively charged molecules, such as RNA and DNA (Cao et al., 2019). Chitosan-NPs of different sizes were explored as delivery system to murine BM-derived HSPCs (Zaki et al., 2015). The authors found that high concentrations of chitosan-NPs affected cell viability of mouse BM cells, in particular for small (200 nm) sized NPs. At low concentration, medium-sized NPs reduced the percentage of HSCs, while intermediate and high concentrations reduced the viability specifically of myeloid committed progenitors, indicating size- and concentration-dependent cytotoxic effects of chitosan NPs. NPs made of high molecular weight chitosan increased the cytotoxicity towards human peripheral blood mononuclear cells (PBMCs) (Jesus et al., 2020). Despite its biodegradability, chitosan also possesses immunostimulatory properties (Han et al., 2016). Thus, further investigation is needed to evaluate whether chitosan-NPs are a suitable delivery system for human HSPCs.

## 2 Monitoring of hematopoietic stem and progenitor cells by nanoparticles for noninvasive imaging

HSC transplantation represents the major curative strategy for numerous malignant and non-malignant hematopoietic diseases and is performed routinely in clinical practice (Howard et al., 2015; Laberko and Gennery, 2018; Staal et al., 2019). Migration of transplanted cells to the bone marrow niche (‘homing’) is an important prerequisite for treatment success. Monitoring of this process helps to identify impaired homing early after transplantation, allowing to intervene to improve engraftment efficacy and transplantation outcome. Viral vectors have been used for cell marking and tracking, but quiescent HSCs are difficult to label by this strategy. AdV vectors only transduce cells that undergo mitosis (Miller et al., 1990), and LV vectors require metabolic activity for viral integration (Sutton et al., 1999), thus more efficient methods are needed to allow marking of quiescent cells. To regard HSC labeling and detection as feasible, several circumstances must be met. Firstly, the detection method should be sensitive enough to monitor labeled cells *in vivo*. Secondly, the labeling should be biocompatible and preserve the viability and functionality of transplanted HSCs. Thirdly, the cell-label association should be stable to track HSCs over a longer period of time. Due to a lower risk of label detachment, intracellular labels delivered by NPs may be preferred over surface-labeling. Labeling with NPs enabled the tracking of the biodistribution of HSPCs using noninvasive biomedical imaging, such as fluorescent imaging, magnetic resonance imaging (MRI) and magnetic resonance spectroscopy (MRS).

### 2.1 *In vitro* labeling procedures and imaging of hematopoietic stem and progenitor cells

The first studies combining NPs and HSPCs explored *in vitro* labeling procedures to provide tools to monitor homing and engraftment of transplanted HSCs. Superparamagnetic iron oxide (SPIO) and other intrinsically monitorable NPs, including gadolinium oxide-NPs, mesoporous silica-NPs, and PLGA-NPs encapsulating fluorine 19 (19^F^) as contrast agent for MRI and MRS, were utilized as labelling agents for HSPCs (Table 1). Cord blood HSPCs were labeled with differently sized NPs or liposomes made of SPIO ferumoxide, ultrasmall SPIO (USPIO) ferumoxtran, transferrin-coated magnetic polysaccharide, P7228 (second-generation USPIO) and gadopentetate dimeglumine (Daldrup-Link et al., 2003). While all NPs were non-toxic and suitable for HSPC labeling, SPIO NPs with a diameter of about 100–150 nm were more efficiently targeted to HSPCs than monocrystalline iron oxide and USPIO NPs, which have diameters of 20–40 nm. Similarly, England et al. studied the uptake of SPIO by CD34^+^ HSPCs in combination with the transfection agent protamine sulfate (a drug used to reverse heparin anticoagulation), both approved agents for use in patients (England et al., 2013). They found that the uptake of ferumoxide by human HSPCs was enhanced after exposure to protamine sulfate. SPIO labeling of CD34^+^ cells did not affect cell viability and labeled HSPCs could be visualized *in vitro* by 3T MRI scanning. Similar, another strategy employed nanocomplexes of ferumoxide and protamine sulfate for non-invasive monitoring of CD34^+^ HSPCs by MRI (Arbab et al., 2005). Labeling of HSPCs with ferumoxide-protamine sulfate complexes did not induce cellular toxicity or affect SDF-1 induced migration and their ability to form HPCs.

Bregoli et al. studied the toxicity of seven metal and metal oxide NPs between 20–210 nm in size on BM CD34^+^ HSPCs (Bregoli et al., 2009). Analysis of colony-forming unit cultures of CD34^+^ HSPCs incubated with different types of NPs showed that antimony oxide (Sb_2_O_3_) NPs and cobalt NPs had toxic effects, while the other NPs were non-toxic at 5, 25 and 100 μg/ml. Interestingly, they found that Co NPs showed toxicity towards erythroid and granulocytic–monocytic precursors, while Sb_2_O_3_ NPs were specifically toxic to erythroid colony development, suggesting selective toxicity towards different HSPC subpopulations (Bregoli et al., 2009).

In another approach, Duinhouwer et al. labeled cord blood CD34^+^ HSPCs with PLGA-NPs containing 19^F^ (Duinhouwer et al., 2015). NP-loaded CD34^+^ HSPCs were detectable by MRS *in vitro* under physiological conditions. Importantly, the labeling did not affect cell viability and labeled CD34^+^ HSPCs maintained their capacity to proliferate and form different types of progenitor colonies in methylcellulose assays. In a similar approach, HSPCs were labeled with PLGA-NPs containing perfluoro-1,5-crown ether and imaged by MRI (Aday et al., 2014). While the NPs did not decrease cell viability, Aday et al. demonstrated that these NPs modulated the paracrine activity of HSPCs by decreasing the secretion of pro-inflammatory cytokines and attenuating the activity of toll-like receptor 6 and 7. Thus, these NPs not only provided a contrast agent for MRI, but also showed immunomodulatory properties.

### 2.2 *In vivo* tracking of NP-labelled hematopoietic stem and progenitor cells by noninvasive imaging

In 2005, Daldrup-Link et al. were the first to monitor human HSPCs, loaded with SPIO (ferumoxide) NPs or P7228 liposomes, after intravenous injection in BALB/c mice (Daldrup-Link et al., 2005). They found that ferumoxides were taken up by more mature CD34^−^, but not by CD34^+^ cells, while P7228 liposomes were taken up by both CD34^−^and CD34^+^ cells. MRI analysis confirmed that iron oxide–labeled human HSPCs successfully homed to recipient organs, such as the liver and spleen at 1, 4, 24 and 48 h, and the BM at 24 and 48 h after injection, with signal intensities significantly stronger than in controls injected with pure contrast agent.

In addition, multimodal PEGylated mesoporous silica NPs loaded with gadolinium oxide and a fluorescent probe were employed for MRI tracking of early HSPC homing in mice (Sweeney et al., 2018). Uptake of biocompatible mesoporous silica NPs by HSPCs did not affect cell viability. NP-labeled HSPCs were tracked in different hematopoietic compartments and confirmed engraftment in the BM 6–9 days post injection. Interestingly, the authors observed that the majority of cells that had taken up mesoporous silica NPs resembled HPCs, which could be distinguished into two morphologically distinct subpopulations with distinct uptake behavior, illustrating the heterogeneity of CD34^+^ cell populations.

Maxwell et al. developed a multimodal approach for labeling and analysis of engrafting human HSPCs using fluorescent molecules covalently linked to dextran-coated iron oxide NPs, allowing MR and fluorescence imaging (Maxwell et al., 2008). The fluorescent label allowed to enrich NP+ HSPCs by fluorescence-activated cell sorting prior to transplantation, as well as monitoring of NP+ cells *in vivo*. Both quiescent and cycling HSCs were efficiently labeled, without inducing toxicity *in vitro* or *in vivo*, which permitted the dynamic tracking of repopulating HSCs during the initial weeks after transplantation (Maxwell et al., 2008).

## 3 Nanoparticles to modulate hematopoietic stem cell signaling

Activation of the Wingless (Wnt) pathway is important for the self-renewal and expansion of HSPCs. Despite some controversies on the role of Wnt proteins and Wnt regulatory factors in the HSC niche, *ex vivo* culture of murine HSCs in the presence of Wnt5A improved their repopulation potential during transplantation experiments (Nemeth et al., 2007), and Wnt3a proteins are known to increase murine HSC self-renewal *ex vivo* (Reya et al., 2003). Without sufficient addition of the detergent CHAPS, purified hydrophobic Wnt proteins rapidly aggregate and lose their activity, but CHAPS interferes with the self-renewal potential of HSCs. To circumvent this problem, Wnt proteins can be encapsulated inside PLGA-NPs and liposomes to maintain their stability over prolonged periods of time in stem cell culture systems (Tuysuz et al., 2017). In another approach, pharmacological activation of the Wnt signaling pathway increased the self-renewal capacity of HSCs. Glycogen synthase kinase 3β (GSK-3β) is a dominant regulator of HSC function due to its capacity to inactivate the Wnt–β-catenin pathway (Ko et al., 2011). Repeated high-dose inhibition of GSK-3β in transplant recipients has convincingly demonstrated to enhance the repopulation kinetics of engrafted HSCs (Trowbridge et al., 2006). Based on this finding, Stephan et al. developed a NP-based adjuvant delivery approach to improve outcomes of HSC transplantations (Stephan et al., 2010) by formulating liposomes and liposome-like synthetic NPs encapsulating the GSK-3β inhibitor TWS119, harboring a phospholipid surface layer including thiol-reactive maleimide headgroups. The NPs were covalently surface linked to CD34^+^ HSPCs *via* abundant free thiols in the plasma membrane. The multilamellar lipid NPs slowly released the GSK-3β inhibitor over 7 days and significantly enhanced reconstitution by the HSC graft, without compromising the homing properties of donor HSCs or their multilineage differentiation potential. For development of clinical applications, elucidation of the long-term consequences of prolonged NP retention on the HSC surface is needed.

### 3.1 Improving hematopoietic stem cell homing through nanoparticles

**FIGURE 3:**
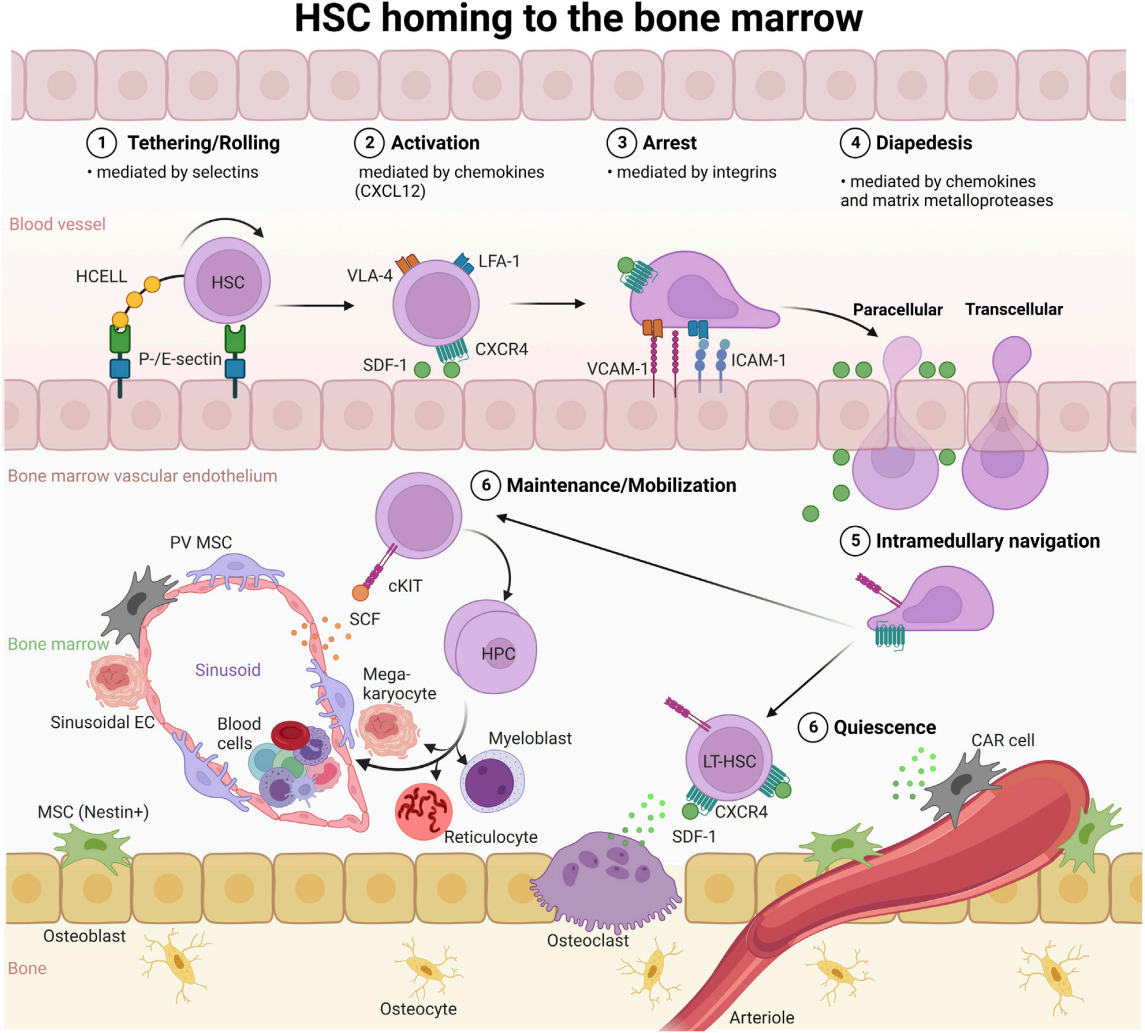
HSC homing to the BM. HSC homing, as in the context of HSC transplantation, depends on cell adhesion molecules (selectins and integrins) and activation of the SDF-1 (CXCL12)/CXCR4 axis: 1) Selectins facilitate the initial tethering/rolling of HSCs on ECs, followed by 2) chemokine-induced activation of integrins on the HSC surface, and 3) firm adhesion (arrest) of HSCs on the endothelium *via* integrin-integrin ligand (LFA-1:ICAM-1, VLA-4:VCAM-1) interactions. Subsequently, 4) HSCs undergo diapedesis preferentially through the EC body, designated as transcellular transmigration, or extravasate *via* the paracellular route through BM ECs and reach the BM niche. 5). *Via* intramedullary navigation, HSCs migrate to and lodge in the endosteal and vascular niches. HCELL, hematopoietic cell E-/L-selectin ligand; VLA-4, very-late antigen-4; LFA-1, lymphocyte function-associated antigen-1; ICAM-1, intercellular adhesion molecule 1; VCAM-1, vascular cell adhesion molecule; SCF, stem cell factor; SDF-1, stromal-derived factor 1; CAR, CXCL12-abundant reticular; PV, perivascular stromal; EC, endothelial cell; MSC, mesenchymal stroma cell.

The main goal of HSPC targeting is to confer to LT-HSCs the ability to sustain normal hematopoiesis throughout life. Several strategies have been employed to improve homing and engraftment of transplanted HSCs for malignant and non-malignant hematological diseases. Much research focuses on the adhesion cascade that precedes the extravasation of HSCs to the BM (Figure 3). Current studies focus on increasing the ability of HSCs to sense CXCL12 (also known as SDF-1) gradients *via* the CXCR4 receptor *in vivo*, the modulation of selectin signaling between HSCs and the endothelium, metabolic modification of HSC homing properties, e.g., through inhibition of heme oxygenase 1 or GSK-3β, or enhancing the availability of chemotactic factors. For example, transplantation outcomes can be improved by the concurrent delivery of small-molecule drugs that promote the homing capacity of HSCs to the BM (recently reviewed (Chander and Gangenahalli, 2020a)). In this context, NPs have been employed as delivery systems to release factors that boost hematopoietic reconstitution in the course of HSC transplantation (Stephan et al., 2010; Huang and Liu, 2012). Chemokine releasing NPs, for example chitosan NPs loaded with SDF-1, have also been developed and evaluated *in vitro* (Huang and Liu, 2012), and have the potential to regulate HSC homing and mobilization *in vivo*, given that they could be delivered specifically to the BM niche.

## 4 The bone marrow niche and hematopoietic stem cell homing

While some HSCs circulate in peripheral blood or stay in other hematopoietic sites such as spleen and liver, the majority localizes to the BM, the major hematopoietic tissue after birth. The BM represents a complex microenvironment made up of different cellular components, which have been the subject of a large body of research (for recent reviews (Ashok et al., 2021; Fröbel et al., 2021)). HSC quiescence, proliferation, differentiation and migration are constantly adapted to systemic needs and controlled by non-hematopoietic BM niche cells, which relay circulating messenger and neuronal signals from outside the BM to HSPCs (Zhang et al., 2003; Sipkins et al., 2005; Katayama et al., 2006; Butler et al., 2010; Mendelson and Frenette, 2014; Morrison and Scadden, 2014). Hence, an estimated 10,000 HSCs and millions of HPCs reside within the human BM and release billions of blood cells into the circulation every day (Catlin et al., 2011). Different HSC niches, including endosteal and vascular (arteriolar and sinusoidal), have been described within the BM (Zhang et al., 2003; Méndez-Ferrer et al., 2010; Birbrair and Frenette, 2016; Tamma and Ribatti, 2017; Fröbel et al., 2021) (Figure 3).

In the mouse BM, most HSCs are present in perivascular locations within 10 µm distance from the endothelium in close contact with either sinusoids or arterioles (Ding et al., 2012; Kunisaki et al., 2013; Nombela-Arrieta et al., 2013). Deeply quiescent HSCs are thought to reside around arterioles close to the endosteum, while the more abundant activated HSCs are believed to localize in the vicinity of niche-spanning sinusoidal vessels at the interface to the circulation, ideally positioned to sense microenvironmental changes in order to meet the demand for new blood cells (Xie et al., 2009; Ehninger and Trumpp, 2011; Pietras et al., 2011; Kunisaki et al., 2013; Nombela-Arrieta et al., 2013).

Different cell types have been identified in proximity to murine HSCs *in vivo*, including endosteal osteoblasts, sinusoidal ECs, leptin receptor-positive (Lepr+) perivascular stromal cells, CXCL12^high^ reticular cells, nestin+ mesenchymal stem cells, non-myelinating Schwann cells, regulatory T cells and megakaryocytes (Kumar and Geiger, 2017; Perlin et al., 2017), which secrete growth factors and cytokines that regulate HSC quiescence, homing and differentiation. For example, osteoblasts secrete a variety of cytokines, including osteopontin, angiopietin-1 and -3, thrombopoietin, granulocyte colony-stimulating factor, stem-cell factor (SCF) and SDF-1 (Kumar and Geiger, 2017; Perlin et al., 2017), which regulate HSC self-renewal and homing.

Homing and engraftment of intravenously administered HSCs *via* the blood to the BM HSC niche is enforced by HSC-attracting chemotactic and other bioactive molecules released in the BM microenvironment (Heazlewood et al., 2014; Ratajczak and Suszynska, 2016). The chemotactic factors include SDF-1 and SCF (Peled et al., 2000; Xu et al., 2018). Endothelial and stromal cells are an important source of SDF-1 and SCF, which promote HSC maintenance and localization to the perivascular BM niche (Kumar and Geiger, 2017; Perlin et al., 2017). For example, Tie2-Cre-mediated inactivation of SDF-1 and SCF in ECs leads to HSCs depletion in the BM niche (Kisanuki et al., 2001; Sipkins et al., 2005; Greenbaum et al., 2013; Morrison and Scadden, 2014).

ECs are important for the initial steps of the homing process, which involves receptors that facilitate HSC tethering and rolling along the endothelium under physiological shear flow conditions (Peled et al., 1999) (Figure 3). The initial tethering process is mediated by selectins, a class of adhesion receptors. Outside the BM, ECs upregulate the expression of E- and P-selectin during inflammation to attract leukocytes (Mazo et al., 1998). In contrast, BM ECs constitutively express selectins (Frenette et al., 1998; Mazo et al., 1998). Human CD34^+^ HSCs express several selectin ligands, including PSGL-1 (P-selectin glycoprotein ligand-1) and HCELL (hematopoietic cell E-/L-selectin ligand) (Dimitroff et al., 2001), which mediate tethering and rolling along the BM vasculature (Figure 3). During the rolling process, HSCs encounter the chemokine SDF-1. Chemokine receptor signaling then activates the integrins VLA-4 and LFA-1 to bind to their ligands VCAM-1 and ICAM-1, respectively. VCAM-1 and ICAM-1 are expressed on the BM vasculature and mediate firm adhesion of HSCs and ECs (Peled et al., 1999; Peled et al., 2000). This is followed by endothelial transmigration, which is also mediated by VLA-4 and LFA-1. Interestingly, a recent proteomics screen identified CD34 itself, which is expressed on HSPCs and ECs, as a selectin ligand that plays a role in the first steps preceding homing of HSCs to the BM (Figure 3) (AbuSamra et al., 2017). Upon extravasation, HSCs migrate through extravascular space and lodge in the BM niche (Heazlewood et al., 2014; Perlin et al., 2017). Thus, the BM vasculature and its endothelium are more than a passive border, rather, they play an active part in the homing of HSCs to the BM niche.

### 4.1 Nanoparticles targeting the bone marrow niche

Successful delivery of NPs and their cargo to the BM niche *in vivo* requires knowledge of the various physiological barriers and their patho-physiological state in health and disease, and understanding both passive and active targeting strategies (He et al., 2021). Passive delivery refers to NPs in the bloodstream that can localize in tissues through passive diffusion, as for example in neoplastic tissues by the enhanced permeability and retention (EPR) effect, which is facilitated by alterations in the lymphatic and vascular vessel structure, or in the liver promoted by the discontinuous vasculature in hepatic sinusoids (Augustin and Koh, 2017). Alternatively, NPs can be functionalized with targeting moieties, such as monoclonal antibodies (or their fragments), proteins or peptide-based molecules, nucleic acids (i.e. aptamers), and a variety of small molecules (Sanna and Sechi, 2020). Such targeting ligands not only provide improved affinity and precision towards target cells and tissues, but also increase the cellular uptake of NPs through the cellular endocytosis machinery.

NPs are commonly administered through systemic injections and experience a wide range of flow velocities in the blood. ECs line blood vessels and have been shown to take up NPs from the circulation, but with increased flow rates the NP uptake decreases (Chen et al., 2020). A commonly used strategy to increase the NP half-life in the blood circulation and to prevent rapid kidney clearance and phagocytosis is the surface modifications of NPs with poly (ethyleneglycol) (PEG) or membrane coatings (Cruz et al., 2010; Cruz et al., 2011; Suk et al., 2016).

To date, different components of the BM microenvironment have been targeted by NPs *in vivo*, including BM sinusoidal ECs, osteoblasts, osteoclasts, mesenchymal cells or immune cells. To facilitate localization to the BM niche, NPs with physicochemical characteristics that intrinsically favor bone and BM targeting, or linking of targeting ligands (peptides, membrane coatings, aptamers or small molecules) to the NP surface have been employed (Pang et al., 2013; Galletti et al., 2016; Cheng et al., 2017).

BM ECs are important target cells for various reasons. They signal to other cells in the BM microenvironment, such as pericytes, immune cells, and HSCs (Morrison and Scadden, 2014). For an effective recognition by BM ECs, NPs with intrinsic binding capacity have been described. For example, Sago et al. combined NPs, siRNA and DNA barcoding to screen hundreds of lipid NP formulations of varying size and PEG composition for their *in vivo* binding and silencing capacity towards murine BM ECs (Sago et al., 2018). Interestingly, they found that NP size (20–200 nm) was not a critical determinant for BM EC tropism. Rather, modification of the PEG structure and addition of cholesterol improved targeting to BM ECs (Sago et al., 2018). They identified “BM1” as the first lipid-PEG NP to efficiently deliver siRNA and sgRNA to BM ECs *in vivo*.

Krohn-Grimberghe et al. screened polymer–lipid NPs for their intrinsic bone-binding capacity (Krohn-Grimberghe et al., 2020) by creating a library of hybrid polymer–lipid NPs, surface-modified with siRNA and PEG–lipid conjugates using a high-throughput microfluidic mixing chamber. They further modulated the PEG surface coating by adjusting the molecular weight of the PEG, the length of the lipid chain that anchors PEG on the NP-surface and the PEG surface density. They obtained a NP (“NicheEC-15”) with superior binding avidity to BM ECs. NicheEC-15 NPs encapsulating siRNA were systemically administered in mice to silence genes in BM ECs. The encapsulated siRNA sequences successfully targeted SDF-1 or monocyte chemotactic protein 1 (MCP-1) mRNA and enhanced (when silencing SDF-1) or reduced (when silencing MCP-1) the release of HSPCs and of leukocytes from the BM. This strategy allowed the regulation of HSC release from the BM and could be used to fine-tune hematopoietic processes for therapeutic applications. The authors hypothesized that a denser PEG surface coat of NicheEC-15 might protect the NPs from first-pass entrapment by lung ECs and increase the blood circulation time and uptake into BM endothelium.

In an attempt to increase the localization of PLGA-NPs in the BM, NPs were surface modified with the bisphosphonate alendronate, a calcium-ion chelating molecule that deposits in bone tissue thereby preventing bone loss. The resulting alendronate-conjugated polymer PLGA-PEG NPs displayed increased circulation times and superior binding capacity to bone *in vivo* (Swami et al., 2014). Similarly, alendronate-modified liposomal NPs carrying the SDF-1 gene to increase mesenchymal stem cell recruitment for bone generation were evaluated for targeted drug delivery. Systemic infusion of Aln-Lipo-SDF-1 NPs led to accumulation of NPs in osseous tissues, expression of SDF-1 in osteoblastic cells and attraction of mesenchymal stem cells for tissue regeneration (Chen et al., 2018).

Another approach to increase homing of transplanted HSPCs to the BM presents the *ex vivo* incubation of HSPCs with peripheral blood platelet-derived extracellular microvesicles (pMVs), such as present in a hematopoietic graft. These pMVs are submicron-sized heterogeneous particles released upon platelet activation (Janowska-Wieczorek et al., 2001). Mechanistically, it has been reported that pMVs harbour several homing receptors, such as CXCR4, CD41, CD61, CD62P, PAR-1 and GPIa/Iia, which can be transferred onto HSCs upon pMV binding and assist in the homing and engraftment process of HSCs to the BM (Janowska-Wieczorek et al., 2001). Especially, CXCR4 receptors were abundant on the HSC cell surface after pMV binding and facilitated the interaction with their ligand SDF-1 present on BM sinusoidal endothelium. Based on this finding, HSPCs were isolated from murine BM or human umbilical cord blood and pre-incubated with pMVs. These HSPCs engrafted much faster after transplantation into normal or immunodeficient mice (Janowska-Wieczorek et al., 2001). In contrast to HSPCs aspirated from BM, HSPCs isolated from mobilized peripheral blood are already rich in pMVs as a consequence of platelet activation in the plastic tubing during leukapheresis. This may explain the differences in engraftment kinetics between peripheral blood-derived and BM-aspirated HSPCs (Janowska-Wieczorek et al., 2001; Chander and Gangenahalli, 2020a).

More recently, Chander and Gangenahalli revealed that complexes between pMVs and pluronics form “nanoclouds” that cover single HSCs and increase the migration of HSCs across sinusoidal ECs to the BM (Chander and Gangenahalli, 2020b). Pluronics represent a group of thermoreversible copolymers which can firmly associate with pMVs (Zhong et al., 2018) and have been frequently used for drug delivery approaches. In a study from the early 90s, coating of polystyrene NPs with Pluronic F-127 directed more than 50% of intravenously infused polystyrene NPs to the BM (Porter et al., 1992). Loading of HSCs with pMV nanoclouds, in particularly with PF127-stabilized chitosan-alginate, prior to transplantation into lethally irradiated mice led to a 10-fold increase in homing, and effective engraftment and regeneration of the blood lineages (Chander and Gangenahalli, 2020b).

**FIGURE 4:**
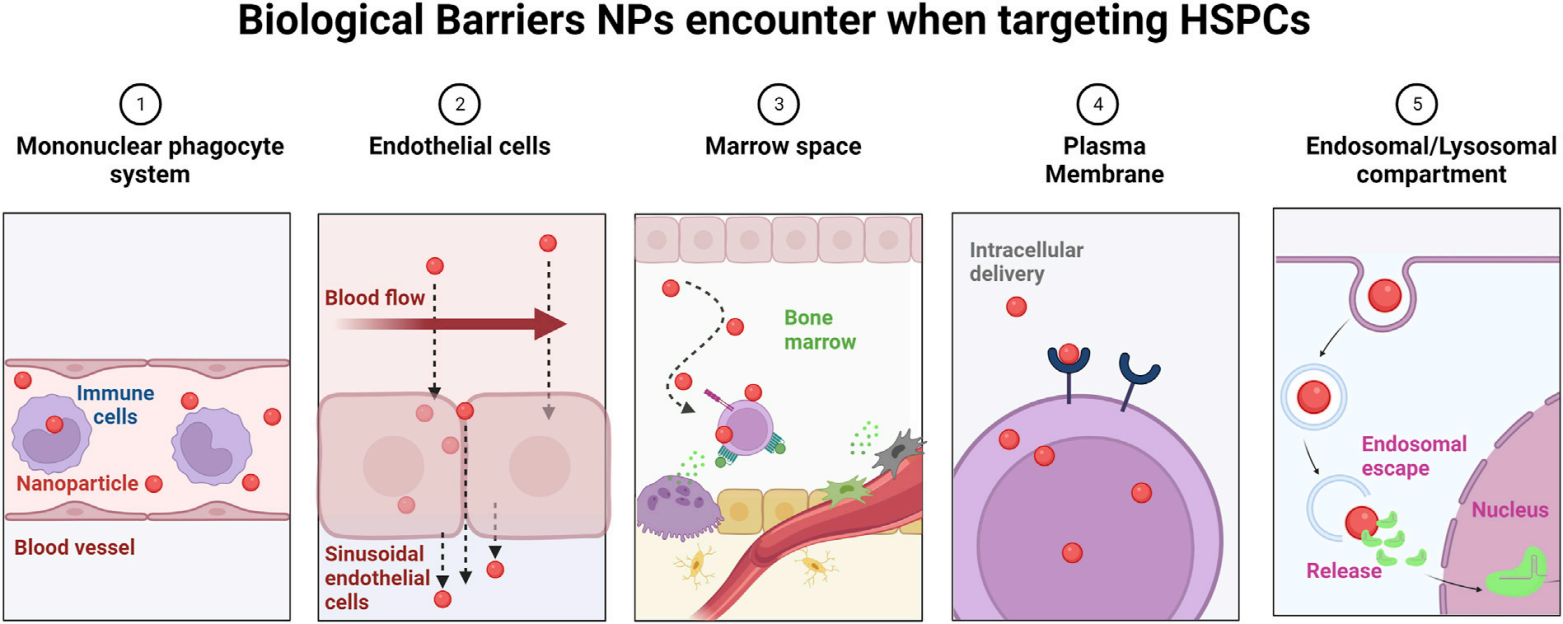
Biological barriers encountered by NPs en route to the BM. Upon intravenous administration, NPs encounter a number of sequential obstacles hindering efficacy and BM-specific delivery. 1) NPs undergo opsonization and subsequent uptake by the mononuclear phagocyte system. This results in nonspecific distribution of NPs in healthy organs. 2) Under normal flow conditions in blood vessels, NPs preferentially localize distant to ECs, limiting both active targeting and passive targeting strategies (e.g., EPR). 3) In the BM space, the blood flow is greatly reduced. To ensure efficient NP uptake, NPs need to get in close proximity to HSCs. 4) The plasma membrane and cellular internalization represent additional barriers for NP uptake by HSCs, and the presence of targeting moieties on the NP surface can affect the uptake mechanism (e.g., clathrin *versus* caveolin) and intracellular routing of NPs. 5) Upon uptake, NPs need to rapidly escape the endosomal route, where NPs are subjected to a low pH environment and enzymes, which prove to be detrimental to the NP payload, especially to genetic material, which is prone to degradation.

To target HSCs in peripheral blood and/or the BM, NPs need to overcome several biological barriers (Figure 4). First, they need to escape recognition by the mononuclear phagocyte system and leave the blood stream by tethering to the ECs, similar to HSCs during the homing process. In this context, it was shown that under normal flow conditions, NPs migrate away from the endothelial surfaces (Blanco et al., 2015), however, surface modification with EC ligands led to partial resistance to flow effects and increased uptake by ECs (Chen et al., 2020), suggesting that surface-modification with EC-ligands promotes NP binding to the BM sinusoidal ECs. Secondly, NPs need to cross the sinusoidal EC layer to reach the BM. Distinct types of ECs form vessels with distinct characteristics. For example, noncontinuous ECs with vascular fenestrations and transmembrane pores measuring 50–100 nm are present in the liver (Braet et al., 2007), while the range of inter-EC slits in the spleen ranges from 200 to 500 nm (Chen and Weiss, 1973). However, pores in sinusoidal blood capillaries have a maximum size of 60 nm, preventing the accumulation of NPs larger than 60 nm in the BM (Sarin, 2010). Instead, intercellular and intracellular transport mechanisms likely play a role in the localization of NPs in the BM. Transcytosis, a poorly understood but yet important mechanism of trans-endothelial transport can be enhanced by the use of targeting ligands (Liu et al., 2019). Thirdly, NPs need to cross the BM space to traffic efficiently to deeply quiescent LT-HSCs. This process might be facilitated by the fact that HSCs are highly motile cells that move in the perivascular spaces, as recently shown by intravital imaging of the BM niche (Upadhaya et al., 2020). Finally, NPs need to be efficiently taken up by HSCs and to escape the endosomal/lysosomal route to release their cargo into the cytoplasm (Figure 4).

In addition, to design NPs that can efficiently modulate HSCs in their natural niche, it is critical to define the hallmarks of the homeostatic healthy steady state niche, as well as during stress conditions and disease. For example, it has recently been observed that hematological diseases have a great impact on the composition and architecture of the BM niche. In a humanized mouse model of SCD, the bone vascular network was found to be disorganized and structurally abnormal, with plenty of highly tortuous arterioles filling the majority of the BM cavity, as well as fragmented sinusoidal EC vessels packed with aggregates of erythroid and myeloid cells (Park et al., 2020). Increased angiogenesis, changes in vessel density and/or infiltration of inflammatory cells are also observed in BM of patients suffering from almost all types of hematological malignancies, similar to the EPR effect observed in solid tumors (Deshantri et al., 2018). How this affects the delivery of NPs to the BM niche needs more research.

Targeting of HSCs in peripheral blood and/or the BM after intravenous injection was also reported by scientists at Intellia (Intellia Therapeutics, I., 2021). They developed an *in vivo* approach to edit the *HBB* gene in HSCs in the murine BM. In this non-viral method, Cas9 mRNA and sgRNAs were delivered directly to target cells by lipid NPs. A similar formulation of lipid NPs has previously been used to edit the mouse transthyretin (*Ttr*) gene in the liver, leading to 97% reduction in serum transthyretin levels that persisted for at least 12 months (Finn et al., 2018). The lipid NPs were validated *in vivo* to identify specific chemical compositions that resulted in enhanced delivery of genetic material to the animals’ BM and HSCs. A week after injection of high doses of CRISPR lipid NPs into the tail vein of healthy (wild type) mice, over 40% of gene editing was found in the entire BM, and in HSCs at levels predicted to have a curative effect in SCD patients. The editing levels were sustained at 5% in the HSC population after 1 year and increased with multiple successive treatments. Importantly, BM transplantation demonstrated no impact on the repopulation and hematopoietic progenitor potential of genetically modified HSCs. These results will now be confirmed in non-human primates.

In the case of hematological malignancies, NP homing to the BM has been achieved by using specific peptides that bind adhesion molecules overexpressed on BM ECs, such as αvβ3 integrins. To this end, liposomes have been coated with Arg-Gly-Asp (RGD) peptides to deliver siRNA or doxorubicin (Schiffelers et al., 2003; Schiffelers et al., 2005). Another common example of endothelial targeting is very late antigen-4 (VLA-4). Liposomes conjugated with a cyclic pentamer peptide, called VLA-4 peptide, have been employed to target hematological malignancies (Ashley et al., 2014). Similarly, targeting BM by conjugating a thioaptamer that is specific to E-selectin to PEG-polylactic acid micelles increased BM accumulation in acute myeloid leukemia (Zong et al., 2016).

### 4.2 Nanoparticles targeting hematopoietic stem cells in the fetal hematopoietic niche

The fetal liver is the major hematopoietic organ during development until shortly before birth. It has been shown that the developing fetus and fetal organs are accessible to NPs (Bongaerts et al., 2020). NPs were transported through the placenta after intravenous or subcutaneous administration to pregnant dams and accumulated in fetal tissues, including brain and liver (Keelan et al., 2015; Manangama et al., 2019). An *ex vivo* study of the barrier properties of human placenta found that polystyrene NPs (50–500 nm) were taken up and able to cross the placenta (Wick et al., 2010). Other studies reported *in utero* localization of NPs after inhalation exposure (Bongaerts et al., 2020). The fetal liver is accessible to *in utero* manipulations, such as HSC transplantation or delivery of gene therapy for the treatment of congenital hematological diseases.

Recently, NPs were applied during *in utero* HSC transplantations (Ricciardi et al., 2018; Loukogeorgakis et al., 2019). One strategy first depleted autologous murine fetal liver HSCs by intrahepatic injections of anti-cKIT antibodies, targeting the SCF-cKIT signaling axis which is critical for HSC survival (Witte, 1990). After depletion of HSCs, liposomes that secreted a GSK-3 inhibitor were covalently linked to the surface of HSCs before *in utero* transplantation. In this NP-assisted HSC transfer, the NPs sustained *in vivo* GSK-3 inhibition, which led to a significant increase in overall cellularity of the post-engraftment HSC donor cell pool, followed by sustained expansion of the donor HSPC populations (Loukogeorgakis et al., 2019).

Novel gene-editing strategies for *in utero* manipulation of HSCs by gene therapy allow for the correction of genes in their endogenous environment. Ricciardi et al. performed *in utero* gene editing mediated by PLGA-NPs that carried triplex-forming peptide nucleic acids (PNAs) and donor DNAs to correct a disease-causing mutation in the β-globin gene locus in a mouse model of human β-thalassemia (Ricciardi et al., 2018). After intra-vitelline vein delivery of fluorescent PLGA NPs encapsulating PNAs, the NPs distributed throughout the mouse fetus with the most prominent accumulation of NPs in the fetal liver. A single dose of PNA/DNA-PLGA-NPs resulted in 8.81% editing frequency of the β-globin gene locus, which led to a sustained correction of anemia, with no detectable off-target mutations.

### 4.3 Nanoparticles targeting hematopoietic stem cells in peripheral blood

While the BM represents the major hematopoietic site in adults, a small fraction of HSCs and HPCs circulates in peripheral blood (Bonig and Papayannopoulou, 2012). Albeit at low frequencies, these HSCs are more easily accessible than HSCs in the BM, and their frequency can be increased by enforced egression (mobilization) by infusion of granulocyte colony-stimulating factor (G-CSF), plerixafor, or the combination thereof (Huntsman et al., 2015; Lidonnici et al., 2017). To confer specificity, NPs can be equipped with targeting moieties (e.g., ligands, antibodies) linked to the NP surface. However, as mentioned above, HSCs lack the expression of unique cell surface markers. Instead, HSCs are characterized by a panel of markers used for positive (CD34 and e.g., CD90, CD133, cKIT) and negative selection (hematopoietic lineage markers and e.g., CD45RA, CD38). Moreover, the marker panel differs among short- and LT-HSCs (Ng and Alexander, 2017). CD34^+^ is still used most widely to enrich for human HSPCs for research or clinical use. For this reason NPs decorated with anti-CD34 antibody have been widely employed in different biomedical applications, including immunomagnetic isolation and enrichment of HSPCs (Liang et al., 2009; Deville et al., 2017; Quynh et al., 2018). Sophisticated isolation of highly enriched HSC populations requires fluorescence-activated cell sorting using multiple markers. Strikingly, megakaryocyte-derived microparticles (MkMPs) possess intrinsic properties to target and bind HSPCs (Jiang et al., 2017). MkMPs are membrane-enclosed vesicles of 0.1–1.0 μm in diameter that are taken up by HSPCs by endocytosis or membrane fusion. Specifically, MkMPs target CD54, CD11b, CD18, and CD43 that are highly expressed in the uropod region of polarized HSPCs during cell migration. The biological function of MkMPs is the transfer of signaling molecules, including proteins, phospholipids and in particular mRNAs and miRNAs (Jiang et al., 2017). An optimized electroporation protocol has recently been developed to externally load MkMPs with genetic material for specific and efficient delivery (up to 81% for an eGFP-encoding plasmid DNA) to HSPCs (Kao and Papoutsakis, 2018). MkMPs possess many beneficial characteristics that could be exploited as delivery system to HSPCs: 1) EVs, such as MkMPs, can easily be generated from donor (autologous or allogeneic) cells (Colombo et al., 2014; Lara et al., 2020); 2) MkMPs have intrinsic capacity to deliver RNA, DNA and protein into HSPCs (Jiang et al., 2017; Lara et al., 2020); 3) MkMPs can be externally loaded with genetic material for gene transfer (Kao and Papoutsakis, 2018); 4) EVs from autologous cells show low immunogenicity and cytotoxicity (Zhu X. et al., 2017); 5) MkMPs can be stored long-term at −80°C without losing biological activity (Jeyaram and Jay, 2017). However, since MkMPs have the capacity to induce differentiation of HSPCs towards the Mk lineage (Jiang et al., 2014), it remains to be determined whether MkMPs can be designed to deliver gene editing tools to HSCs *in vivo*, without inducing a lineage bias. Alternatively, further elucidation of the MkMPs targeting mechanisms could pave the way towards novel HSPC targeting approaches by mimicking the properties that confer HSPC specificity to MkMPs. Another interesting possibility to specifically deliver cargo to HSPCs consists of a hybrid delivery system consisting of NPs coated with MkMP membranes (Powsner et al., 2021). In such an approach, synthetic NPs encapsulating a cargo, such as gene editing components, could be prepared in large batches, and be coated with patient-specific MkMP membranes for targeting of HSPCs *in vivo*. As MkMPs and synthetic NPs have a similar shape and elasticity, and similar size, this suggests that such NPs could be designed to target HSCs.

## 5 NP-based treatment strategies targeting hematopoietic stem cells

NPs hold great promise as delivery system for curative treatments for various genetic, infectious and malignant hematological disorders. With the discoveries of sequence-specific nucleases and their advancement as gene editing tools, new treatment possibilities emerged for hematological patients with unmet medical need (Hacein-Bey-Abina et al., 2014; Hacein-Bey Abina et al., 2015; Sather et al., 2015; Sessa et al., 2016; Kanter et al., 2017; Thompson et al., 2018; Staal et al., 2019). In this paragraph we discuss the possibilities of NP-based therapeutics targeting HSPCs for a variety of hematological disorders.

### 5.1 Hematological disorders and current treatment regiments

The majority of human hematological disorders are caused by mutations affecting HSCs, HPCs or their committed progeny, leading to defects in hematopoiesis or lineage-specific damages. Based on their pathology, they are divided into three main categories: red blood cell disease, white blood cell disease and platelet disease. Commonly known diseases are e.g. sickle cell disease, thalassemia, leukemia, lymphoma and hemophilia. In addition, many rarer hematological conditions exist, including storage disorders, immunodeficiencies and transcriptional syndromes. A range of traditional and highly progressive treatment options are currently used to treat patients with hematological disorders. These include chemotherapy, radiation therapy, targeted biological therapy, BM transplantation, CAR T-cell therapy and gene editing of autologous HSCs in combination with HSC transplantation.

### 5.2 Nanoparticles for the treatment of hematological malignancies

Hematologic malignancies are a broad spectrum of cancers including leukemias, lymphomas, and multiple myelomas that originate from abnormal differentiation of HSPCs in the BM. Consequently, the balance in the BM microenvironment is disturbed, as malignant cells grow at the expense of normal hematopoiesis. Although chemotherapeutic agents are available for the treatment of hematological malignancies, the application of these drugs is restricted due to dose-related toxicity and lack of specificity towards malignant cells. Thus, there is an unmet medical need for suitable drug delivery systems to improve efficacy and safety of treatments for hematologic malignancies. NPs, such as polymeric NPs and micelles, albumin-stabilized NPs and liposomes, have been widely used for the delivery of chemotherapeutic drugs in the treatment of hematological malignancies. Several formulations have been approved for clinical use or are under preclinical development; for a detailed overview we recommend (Deshantri et al., 2018). Most of these formulations target the site of disease manifestation, which in hematological malignancies is predominantly located in the BM and peripheral blood, as well as secondary lymphoid organs, such as the spleen and lymph nodes. Similar to the EPR effect, increased angiogenesis and changes in microvessel architecture, as well as infiltration of inflammatory cells, is seen in BM of patients suffering from virtually all types of hematological malignancies, which likely promotes NP accumulation in these organs. Swami et al. developed alendronate-conjugated PLGA-PEG-NPs loaded with bortezomib, a proteasome inhibitor, for BM targeting (Swami et al., 2014). Another strategy to target malignant cells combines monoclonal antibodies against specific biomarkers with NPs. This strategy is particularly advantageous if the antibody itself exerts cytotoxic effects to the malignant cells. For example, daratumumab, an FDA-approved anti-CD38 antibody effective against multiple myeloma, was coupled to liposomes and combined specificity to the malignant B cells with use as a combination therapy (Deshantri et al., 2018). In another example, doxorubicin-loaded micelles targeted against CD19 were injected in a human acute lymphoblastic leukemia xenograft model. This led to increased survival time compared to control animals (Krishnan et al., 2015). Furthermore, transferrin-targeted doxorubicin-loaded Pluronic85/lipid NPs were developed for the treatment of childhood leukemia (Zhu B. et al., 2017). More recently, biomimetic NPs that imitate naturally occurring structures, such as cell membranes, have been used to deliver therapeutics for the treatment of hematological malignancies. Biomimetic NPs avoid immune recognition and target specific locations in the body by exploiting the unique homing abilities of cellular membranes to deliver cargo, while reducing the risk of toxicity (Powsner et al., 2021). With the advent of CRISPR and its utilization in cancer immunotherapy, new therapies for hematological malignancies are in development. Current approaches mainly focus on the modulation of the T cell receptor and immune checkpoint regulators to increase the T cell response towards malignant cells. In this context, *in vivo* CAR-T cell induction mediated by NPs encapsulating CAR-genes and gene-editing tools have shown promising results in the treatment of leukemia. *In situ* programming of autologous T-cells with the help of NPs could avoid the safety concerns of allogeneic T cells and reduce systemic toxicities (Xin et al., 2022).

### 5.3 Nanoparticles as delivery system of genetic therapy for hematological disorders

Currently, the only available permanent cure for many hematological disorders is transplantation of healthy HSCs which rebuild the hematopoietic system of myelo-ablated patients. But there is a shortage of suitable allogeneic donors and the treatment is linked to the risks of graft rejection and graft *versus* host disease (Cavazzana et al., 2019). The utilization of gene-modified autologous HSCs eliminates the risk of graft *versus* host disease and negates the necessity for immunosuppressive drugs required during allogeneic HSC transplantation. In the past decade, discovery of nucleases that enable site-specific genome editing, such as zinc-finger nucleases (ZFN), transcription activator-like effector nucleases (TALEN), PNAs and clustered regularly interspaced short palindromic repeats (CRISPR)/CRISPR-associated protein nuclease (Cas) (CRISPR/Cas) have emerged as attractive tools to correct or ameliorate diseases or acquired immunodeficiencies, such as those caused by HIV, in autologous HSCs. Overall, the potential of these tools is vast, considering that over 60% of all human disease-causing genetic variants are caused by point mutations (Rees and Liu, 2018). Among these nucleases, CRISPR/Cas systems stand out as they provide a flexible, modular, and cost-effective means to edit the genome.

Gene editing has demonstrated to be beneficial for patients with genetic blood disorders, such as sickle cell disease (SCD). One approach focuses on the repair of the SCD mutation in the HBB gene (SNP rs334, c.20A>T, *p*. Glu7Val). Convincing proof-of-concept data has been obtained using homology-directed repair (HDR) of a Cas9-induced double strand break (DSB) at rs334 (Dever et al., 2016; Uchida et al., 2021a; Uchida et al., 2021b; Lattanzi et al., 2021), and base editing to convert the SCD allele into one encoding Makassar β-globin, a non-pathogenic variant (Newby et al., 2021). Another approach focuses on reactivation of fetal hemoglobin expression based on reducing expression of BCL11A, a transcriptional repressor of the fetal β-like globin genes HBG1 and HBG2 in adult erythroid cells. Depletion of BCL11A in adult erythroid cells reactivates HBG1/2 expression, which is very beneficial for SCD patients. In a recent study in SCD patients, CRISPR/Cas9 genome editing was applied to inactivate the erythroid-specific enhancer of the BCL11A gene (Frangoul et al., 2021), resulting in therapeutic expression levels of γ-globin. Alternative approaches focus on destroying the binding site for BCL11A in the HBG1/2 promoters, either by non-homologous end-joining of Cas9-directed DSBs, HDR of DSBs, or base editing, and have shown promising results in pre-clinical studies (Traxler et al., 2016; Martyn et al., 2018; Metais et al., 2019; Wu et al., 2019; Weber et al., 2020).

Several delivery methods are used to perform CRISPR/Cas9-mediated gene editing in HSPCs, including AAV (Song et al., 2013; Sather et al., 2015) or LV transduction (Traxler et al., 2016), or electroporation of ribonucleoprotein (RNP) complexes, achieving up to 80% efficiency of gene editing in human CD34^+^ HSPCs (Verhagen et al., 2022). If HDR is required, the most effective methods have been electroporation followed by transduction with non-integrating viral vectors (Dever et al., 2016), or concomitant electroporation of RNP complexes and chemically modified single-stranded DNA templates (De Ravin et al., 2017). However, current approaches have several limitations. 1) AAV vectors have a low packaging efficiency and documented immunogenicity, while classical viral vectors carry the risk of insertional mutagenesis (Wu et al., 2010; Yin et al., 2014). Moreover long-term Cas9 expression associated immunogenicity can cause lysis of edited cells, thus further limiting the use of viral vectors (Mehta and Merkel, 2020). 2) While successful non-viral gene-editing in HSPCs using electroporation has been reported (Humbert et al., 2019), this approach remains associated with cellular toxicity (Gundry et al., 2016; Charlesworth et al., 2018). 3) Current gene editing approaches require *ex vivo* culturing and manipulation of HSCs in the presence of cytokine cocktails, which is thought to negatively impact the long-term viability and repopulation capacity of HSCs. 4) *Ex vivo* gene therapy of HSCs needs to be performed in specialized healthcare centers with high costs, restricting patient access. These limitations have inspired the development of *in vivo* delivery systems, such as NPs for gene editing tools, which may overcome the need for *ex vivo* manipulation of patient HSCs, and reduce off-target effects by Cas9 activity (Wilbie et al., 2019). Such developments could bring safe and effective genetic therapies to all parts of the world, including areas of sub-Saharan Africa where the burden of diseases such as SCD and HIV are high (Ndung’u et al., 2019; Cannon et al., 2021).

For efficient gene editing, tools such as CRISPR need to be administered at sufficiently high concentration inside target cells *in vivo* en route to the nucleus. DNA and RNA are by nature prone to degradation by serum nucleases, and possess poor membrane permeability potential (Fu et al., 2014). Lentiviral vectors are not suitable for *in vivo* gene editing, due to rapid complement inactivation after injection and the lack of site-specific targeting motifs (Takeuchi et al., 1994). Several reviews have recently addressed the NP formulations that have been developed to incorporate different types of non-viral gene editing tools (Wilbie et al., 2019; Duan et al., 2021; Naeem et al., 2021; Xu et al., 2021). NPs protect their payload and confer novel physicochemical properties to their cargo, which enables the effective uptake of gene-editing components by the cellular endocytosis machinery. Importantly, as aforementioned, NPs could be equipped with targeting motifs to HSCs in the BM or peripheral blood HSCs. In particular, lipid NPs, polymeric NPs and gold NPs offer great potential as non-toxic delivery system for gene editing components (Lee et al., 2017; Finn et al., 2018; Lee et al., 2018). During the formulation of lipid and polymeric NPs, the gene editing components are encapsulated into the NP core (Cruz et al., 2021), which has the advantage that the RNP complex is protected from degradation during delivery to the target site (Wang et al., 2016). We have recently demonstrated that PLGA-NPs represent a suitable delivery system for co-encapsulated RNP complexes and fluorescent probes, and efficiently edited the HBG1/2 genes in primary human CD34^+^ HSPCs leading to elevated levels of fetal hemoglobin mRNA, without affecting hematopoietic progenitor clonogenic potential (Cruz et al., 2021). In another recent approach, polymeric poly-β-amino ester (PBAE) NPs were used as delivery system for Cas9 RNP complexes to disrupt the CD33 gene in human HSCs, a strategy to protect HSCs from anti-CD33 treatments in acute myeloid leukemia patients (El-Kharrag et al., 2022). Importantly, NP-edited CD34^+^ and CD34^+^CD90^+^ cells showed efficient long-term engraftment in sublethally irradiated NSG mice and retained multilineage differentiation potential.

**FIGURE 5:**
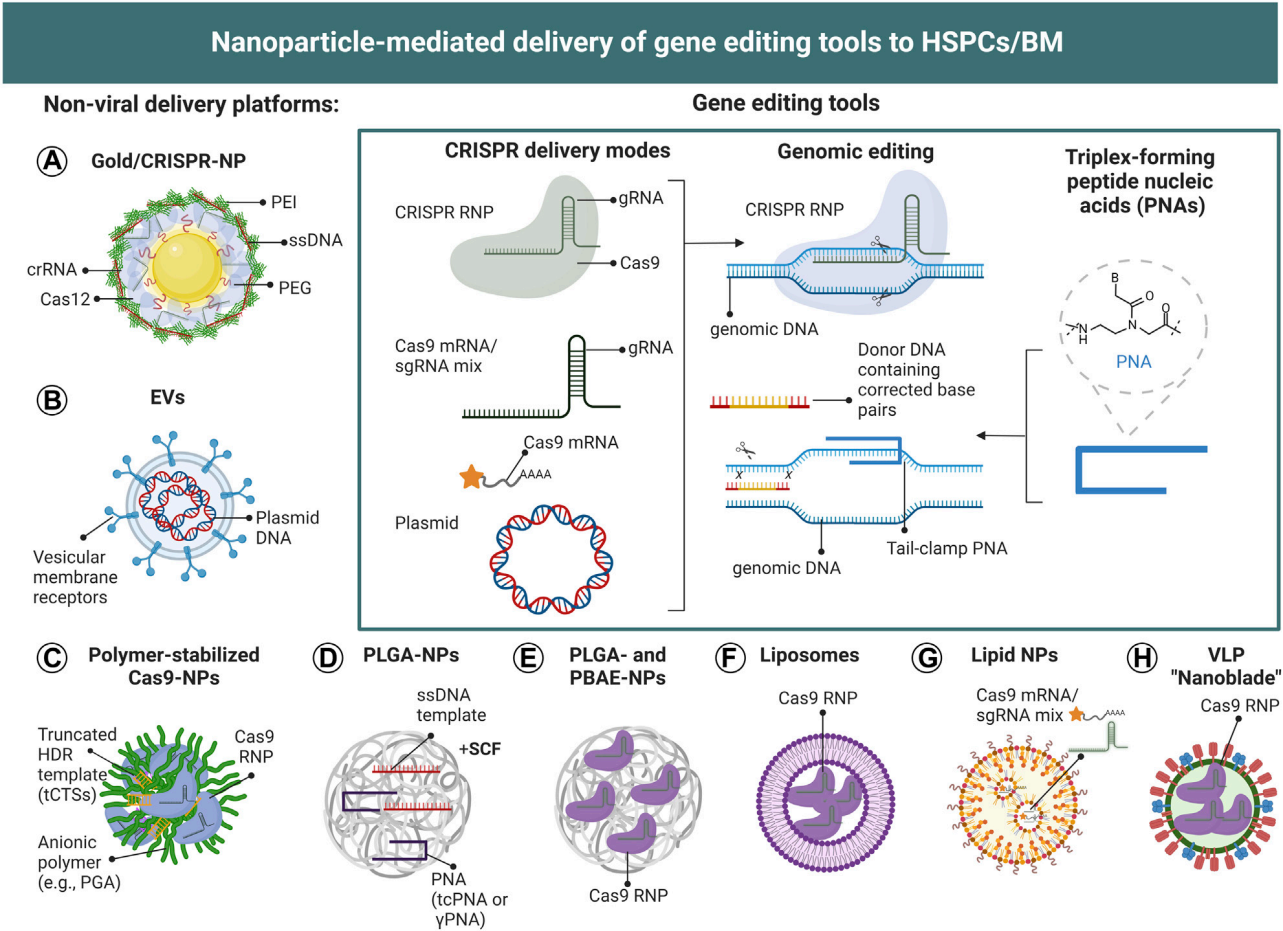
Nanoparticle-mediated delivery of gene editing tools to HSCs. **(A–H)** Schematic illustration of CRISPR- and PNA-based NP-delivery systems employed for the genetic modification of HSPCs. To date, gold NPs, polymer-stabilized NPs, PLGA-NPs, lipid NPs, liposomes, virus-like particles (VLPs) and EVs have been utilized for the genetic modification of HSPCs. (Inset) CRISPR/Cas9 can be delivered as plasmid DNA, mRNA, or RNP complex (together with double-stranded or single-stranded DNA templates in the case of HDR), to achieve site-specific gene editing. The different formats can be encapsulated or surface-deposited for efficient intracellular delivery. Plasmid DNA needs to be delivered into the nucleus and be transcribed into mRNA, which then will be translated into Cas9 protein in the cytoplasm and be transported back into the nucleus to form a CRISPR RNP complex which can exert gene editing function. For mRNA delivery, the payload should be released in the cytosol to enable mRNA translation to protein. In contrast, CRISPR RNP need to be delivered to the nucleus.

In contrast to polymeric and lipid NPs, DNA, RNA and protein are commonly deposited on the surface of metallic NPs, such as gold, *via* surface modification chemistry or charge interactions (Rosi et al., 2006). In particular positively charged NPs, such as gold NPs, allow the deposition of large amounts of genetic material on the NP surface, and therefore represent an attractive nanomaterial for the delivery of gene editing tools` (Lazarus and Singh, 2016). Polymer-stabilized RNP complexes can also form NPs by electrostatic interactions (Nguyen et al., 2020), however, similar to surface-deposited gene editing components, these complexes are not protected from proteases and nucleases, unless further surface functionalization is employed. In Figure 5 we summarize the currently used NP-mediated delivery strategies for the gene editing components CRISPR and PNAs described in this review.

To test the utility of PLGA-NPs for the delivery of gene editing tools to HSPCs, McNeer et al. encapsulated PNAs and donor DNA templates containing a desired sequence modification into the core of PLGA NPs (McNeer et al., 2011). PNAs consist of nucleobases with a peptide-like backbone and enable high-affinity triplex structure formation with DNA, triggering DNA repair and stimulating DNA recombination near the PNA binding site (Rogers et al., 2002). Editing of the HBB gene locus with PLGA/PNA/DNA NPs in CD34^+^ HSPCs led to site-specific modifications of 0.5–1% per treatment without induction of cytotoxicity and proved to be superior over nucleofection. This was the first demonstration of biodegradable NPs as delivery system for genome editing components. Two years later, McNeer et al. demonstrated *in vivo* gene editing of the HIV co-receptor CCR5 (to prevent or cure HIV infection) and HBB gene loci in HSCs by intravenous injection of PLGA/PNA/DNA NPs in a humanized mouse model, albeit at low editing frequency (0.05% in the BM, and 0.43% in the spleen) (McNeer et al., 2013).

A recent study reported *in vivo* HSC gene editing in β-thalassemic mice using intravenously injected PLGA NPs, carrying PNAs and donor DNAs to correct a disease-causing mutation in the β-globin gene locus, in combination with SCF given intraperitoneally prior to NP administration (Bahal et al., 2016). Bahal and McNeer first reported the incorporation of mini-PEG groups at the γ-position of some or all PNA units. *In vivo* treatment in a β-thalassemic mouse model led to a gene editing frequency of almost 4% in total BM cells and 6.9% in HSCs, and improved blood hemoglobin levels lasting for at least 140 days. The authors found that SCF enhanced the PLGA/PNA/DNA NPs-mediated gene editing *in vivo*, likely a result of increased HSC mobilization which may allow more efficient gene transfer. In a mouse model of β-thalassemia, PLGA/PNA/DNA NPs were also applied intra-amniotically at selected gestational ages with no impact on survival or postnatal growth. Deep sequencing revealed correction of the disease-causing mutation in the HBB gene in 6% of all BM cells. This led to a sustained correction of anemia, with no detectable off-target mutations (Ricciardi et al., 2018). While PNAs lag behind CRISPR in gene editing efficiency, they have the safety advantage of low off-target editing and not inducing double-stranded DNA breaks.

Another gene editing approach to treat SCD and β-thalassemia focusses on the introduction of a specific deletion within the HBG1/2 promotor region recapitulating a natural occurring mutation known as hereditary persistence of fetal hemoglobin, which is known to ameliorate disease symptoms (Akinsheye et al., 2011). In this context, Shahbazi et al. developed a multilayer PEGylated gold NP platform functionalized with guide RNA, Cpf1 (or Cas12) endonuclease, polyethylenimine (PEI) and single stranded DNA templates, leading to 8.8% HDR in CD34^+^ HPSCs (Shahbazi et al., 2019). Gene edited CD34^+^ HPSCs engrafted in sub-lethally irradiated immunodeficient mice and showed stable levels of gene editing of 5% in peripheral blood at 22 weeks post transplantation. The authors found gold NPs more efficient for HDR than electroporation, without affecting HSPC viability. Instead, they found a positive effect of gold NPs on the progenitor colony formation potential, with HDR levels initially decreasing after HSC transplantation before eventually stabilizing. This phenomenon was also reported by other groups (Xu et al., 2017); the peak likely illustrates NP uptake and gene editing in mature CD34^+^ HPCs with limited life-span.

Recently, Nguyen et al. reported a method to improve the efficacy of CRISPR/Cas9-based HDR in primary CD34^+^ cells by adding truncated Cas9 target sequences at the ends of the HDR template to interact with Cas9 RNPs and to shuttle the template to the nucleus (Nguyen et al., 2020). In addition, aggregating Cas9/gRNA RNP complexes with polyglutamic acid into NPs of 100 nm further improved editing efficiency to 15% in primary mobilized peripheral blood HSPCs. Polyglutamic acid-stabilized RNP NPs could be lyophilized, enabling upscaling of gene-modified cell manufacturing for research or clinical translation.

Lipid NPs delivering Cas9 mRNA along with a potent single gRNA have also been developed for the treatment of hemophilia, a genetic hematopoietic disorder with spontaneous bleeding caused by loss of gene function in the coagulation pathway (Han et al., 2022). The gRNA was designed to target antithrombin, an endogenous negative regulator of thrombin generation that is encoded by the serpin family CC member 1 (SERPINC1) gene. The lipid NPs successfully delivered CRISPR *in vivo* to the liver. Three consecutive doses resulted in 50% of antithrombin inhibition and enhanced thrombosis, without induction of off-target effects (Han et al., 2022).

A more recent technology utilizes “nanoblades”, consisiting of modified murine leukemia virus or HIV-derived virus-like particles (VLP) fused to RNP complexes (Gutierrez-Guerrero et al., 2021). Gene editing with baboon envelope pseudotyped nanoblades led to 40% edited deletion in the Wiskott-Aldrich syndrome (WAS) gene locus in CD34^+^ human HSPCs, without inducing cytotoxicity. This technology was also combined with donor-encoding rAAV6 vectors, resulting in up to 40% of stable expression cassette knock-in into the WAS gene locus.

Every method of incorporating gene editing components onto NP platforms has advantages and disadvantages, and some are better suited for specific cell types. Table 2 shows an overview of the pros and cons of the most commonly used NP-platforms to deliver gene editing tools to HSPCs.

**TABLE 2:** Overview advantages/disadvantages of NP-based delivery system for gene editing components.

Type of NP	Advantage	Disadvantage	Ref
Gold NPs	-Easy preparation and surface modification	-Not-biodegradable	Lazarus and Singh (2016); Caffery et al. (2019); Ferreira et al. (2020); Sani et al. (2021); Kavanagh and Green, (2022)
	-High linking capacity for genetic material	-Unknown long-term toxicity	
	-Biocompatible	-Aggregation	
	-Tunable size and large surface area	-High costs for large-scale production	
	-Applicable for all types of CRISPR delivery modes	-potentially cytotoxic	
Polymeric NPs	-Easy preparation and tunable surface modification	-Unknown long-term toxicity	Bose et al. (2016); Chen et al. (2019); Duan et al. (2021); Kavanagh and Green, (2022)
	-Large-scale production possible	-Agglomeration	
	-High loading capacity	-Use of organic solvents	
	-Protection of payload from degradation		
	-Low toxicity		
	-Biodegradable		
	-Low immunogenicity		
	-Controlled drug release		
	-Possibility of spatio/temporal release design		
	-Adjustable chemical and physical properties		
	-Excellent stability and long-term storage		
Liposomes	-Easy preparation	-Moderate loading capacity	Caffery et al. (2019); Aguilar-Pérez et al. (2020); Kavanagh and Green, (2022)
	-Low toxicity	-Low stability	
	-Biodegradable	-Agglomeration	
	-Low immunogenicity	-Endosomal degradation	
	-Protection of payload from degradation		
	-Cost-efficient		
	-Can prolong drug half-life		
Lipid NPs	-Biodegradable	-Moderate loading capacity for hydrophilic drugs	Ghasemiyeh and Mohammadi-Samani, (2018); García-Pinel et al. (2019); Dhiman et al. (2021)
	-Biocompatible	-Payload expulsion under storage conditions	
	-Low toxicity	-Spontaneous disintegration (polymorphic transition)	
	-Large-scale production possible		
	-Possibility of controlled drug release		
	-Low immunogenicity		
	-Tunable surface-modification		

## 6 Challenges and opportunities for nanoparticles in the treatment of hematological diseases

Despite recent developments and breakthroughs in the field of NP-mediated delivery of gene editing tools allowing deployment of CRISPR/Cas9 gene editing directly *in vivo* in primates, including humans (Gillmore et al., 2021; Musunuru et al., 2021; Rothgangl et al., 2021; Mullard, 2022), substantial obstacles remain for the delivery of NPs to HSCs *in vivo*. First, several external and internal barriers must be overcome that severely limit site-specific delivery of NPs *in vivo* and consequently affect therapeutic efficacy. In addition, opsonization and subsequent sequestration by the mononuclear phagocyte system represents another challenge leading to nonspecific *in vivo* distribution and accumulation of NPs in healthy organs, such as the spleen and the liver. Thus, NP developers face the challenge to reduce non-specific accumulation and to reach therapeutic levels at target sites. PEGylation can significantly prevent sequestration by mononuclear phagocytes, decrease nonspecific distribution, unexpected immune responses and improve the stability of NPs. However, the downside is the formation of an aqueous phase on the NP surface, which reduces the interaction of NPs with target cells and their ability to escape the endosomal route. This phenomenon is also known as the PEG dilemma: prolonged blood circulation *versus* reduced cellular uptake/endosomal escape. Entrapment in endosomes/lysosomes leads to payload degradation and is a potential failure point of NP systems carrying gene editing tools. Incorporation of pH-sensitive compounds or cleavable chemical linkers between the PEG moiety and the NP surface can overcome entrapment in lysosomes (Schmaljohann, 2006; Zerrillo et al., 2019; Shinn et al., 2022). Upon reduction in pH during the endosomal/lysosomal routing, the linkers can be cleaved to expose a positively charged surface to trigger endosomal escape and translocation to the cytoplasm. Modification of PEGylated NPs with ligands is an efficient way to combine the advantages of PEG with cell-specific delivery. Moreover, the use of antibodies as targeting ligands has the advantage that cloning can be employed to introduce point mutations in the backbone that decrease antibody-dependent cellular cytotoxicity and antibody-dependent cellular phagocytosis by the mononuclear phagocyte system (Kang and Jung, 2019).

To target BM HSCs, NPs have to pass several external barriers (bloodstream-EC, EC-BM, BM-LT-HSCs). This requires the design of versatile NPs carrying multiple properties to tether to the endothelium, pass the EC layer to the BM niche, target LT-HSCs and deliver cargo efficiently. Scientist recently reported efficient delivery of CRISPR by lipid NPs with BM tropism. Gene editing of HSCs in murine BM was observed at levels predicted to be curative for SCD (Intellia Therapeutics, I., 2021). However, the specificity of NP targeting to HSCs remains to be determined.

Because HSC markers, including CD34, are also present on other HPCs and ECs, it is currently not possible to deliver NPs specifically to HSCs using common HSC markers. One strategy to increase binding and uptake by HSCs may be the combination of NPs with bivalent antibodies designed to target multiple HSC motifs with lower affinity, such that a high-affinity interaction between the NP and multiple markers on HSCs would be favored (Husain and Ellerman, 2018). In addition, an improved understanding of HSC biology, based on studies of purified HSCs, will help to determine which receptors represent the most selective targets on HSCs.

A major concern in the application of NPs in living organisms is safety and specificity. A delivery vehicle that can target the desired cells with high-specificity will also limit off-target effects and improve safety. It is unlikely that a single NP formulation will be universally applicable to target exclusively HSCs. However, the incorporation of targeting motifs could greatly increase the intracellular delivery of NPs and their payload to HSCs. As HSCs do not display unique cell surface markers, and the NPs need to cross multiple barriers to reach HSCs, a targeting motif, or combination thereof, must be wisely chosen to limit the complexity of the NP formulation, while increasing BM accumulation and uptake by HSCs. Future NP platforms could be developed to avoid premature payload release by utilizing biomaterials that respond to stimuli specifically present or highly expressed in the BM, combined with HSC targeting motifs to increase specificity towards HSCs.

The success of future genetic therapies greatly depends on advances in genetic engineering and delivery to the target cells. Besides target cell delivery issues, a current limitation in the translation of CRISPR therapies to the clinic concerns the off-target effects of Cas9 nucleases. High-fidelity Cas molecules with reduced unspecific DNA binding in combination with transient delivery systems are required. Cas9 nickases and mutants that reduce non-specific DNA binding have been engineered specifically to overcome this issue (Lino et al., 2018), which is vital for continued development if CRISPR/Cas9 is to realize its promise for the treatment of human diseases. Efficient gene editing while minimizing off-target effects is generally obtained from delivery of the RNP complex rather than plasmid DNA or mRNA (Kim et al., 2014). NPs allow the transient delivery RNP complexes. Importantly, NP systems can easily be adjusted to incorporate new variants of Cas nucleases with improved on-target specificity and reduced off-target effects.

To cure hematopoietic disorders, it requires genetic correction of LT-HSCs to eliminate or ameliorate disease phenotypes in their progeny. For many inherited diseases, correction of a fraction of HSCs is sufficient to reverse disease pathology. In SCD and b-thalassemia, post-transplant follow-ups have shown that mixed hematopoietic chimerism of 10–30% ameliorates clinical disease symptoms (Hsieh et al., 2009; Chaudhury et al., 2017). If NP-mediated *in vivo* editing does not achieve sufficiently high levels of chimerism after one dose, repetitive dosing can easily be performed to increase chimerism (Intellia Therapeutics, I., 2021). The situation would be different for malignant hematological disorders where all cells that drive the disease would have to be targeted successfully. These conditions would therefore be more difficult to treat let alone cure genetically. Alternatively, NP-based approaches could be designed to achieve immune therapy of hematological malignancies.

NP-based diagnostic strategies for monitoring of HSC greatly depend on the HSC targeting potential of the delivery system, without introducing toxicity or affecting their stem cell properties. Several noninvasive NP-based (multi)modalities have been developed to label HSPCs *ex vivo* before transplantation and detect the initial homing and reconstitution patterns of HSPCs within various organ compartments relevant to hematopoiesis, including the early signs of HSPC engraftment in the BM. Improvement in labeling techniques and imaging probes are needed for long-term tracking of HSCs. NPs are ideally suited and widely used for concomitant imaging and therapeutic purposes. The incorporation of imaging probes and contrast agents on gene editing NP platforms during *in vivo* gene editing allows *in vivo* monitoring of NP distribution at the tissue level, and at the cellular level by flow cytometry. While HPCs proliferate and dilute NPs over time, it is expected that NP-probe conjugates will be detected for longer periods of time in quiescent HSCs compared to HPCs, provided that the imaging properties of the NPs are sufficiently effective and stable.

To build an efficient NP platform for the delivery of gene editing tools to HSCs, the interaction between NPs and gene editing components should be strong enough to ensure that the RNP complexes are stable in the bloodstream before cellular internalization. In contrast, after endosomal/lysosomal escape, the RNP complexes should be released from the NPs to diffuse into the cytoplasm, and finally to translocate into the nucleus. Any problem at any step may cause the entire delivery process to fail. A multipurpose CRISPR/Cas9 delivery system has still to emerge. Rather, multiple methods have been described to deliver CRISPR to cells. Every method has its advantages and disadvantages, and some are better suited for specific cell types. Cationic NPs (organic or inorganic) stabilize the CRISPR payload deposited on the NP surface *via* electrostatic interactions, and an organic shell (mainly a lipid layer) is often used to protect the RNP complexes from nucleases. Targeting ligands can be anchored to the shell to mediate interactions with host cells. On the other hand, during the formulation of polymeric and lipid NPs, the RNP complexes are encapsulated into the NP core and therefore protected from premature degradation and clearance by the immune system. Polymeric NPs have the intrinsic advantage that they display a longer shelf life than lipid NPs. In addition, it is vitally important that long-term toxicity studies on safety of the NPs are performed, and if necessary, improvements in NP design are developed to achieve NPs that are non-toxic, non-immunogenic, and highly stable with high cargo delivery efficiency. The great flexibility inherent in the use of NP-mediated genetic therapy allows the selection of the best possible combination of factors for maximum effectiveness.

## 7 Conclusion and future perspectives

Gene editing technologies, which include CRISPR/Cas nucleases and base editors, hold the promise to permanently modify disease-causing genes in patients. Despite the excitement of the new breakthroughs in gene therapy, *in vivo* application of gene editing components is still in its infancy. Nevertheless, several clinical trials of genetic therapy have been completed or are under way. This is expected to significantly increase over the next couple of years and will include many trials for hematological disorders.

Clearly a number of challenges will have to be overcome. Efficiency of delivery will have to be improved, in particular for the treatment of malignant hematological disorders, such as leukemias where most if not all of the leukemic cells have to be modified or depleted. Specificity remains an issue because cell surface targets are shared between different cell types and hence for example antibody-mediated NP delivery will be a cause of concern when other (non-targeted) cells are also modified as this may change their function, or if they are abundant will require an unfeasibly high NP dose for treatment. Specificity of the editing system itself will also have to be improved further to ensure that sequences other than the intended target sequences are not modified.

If targeting efficiency of LT-HSCs or leukemic stem cells would be too low, repeated treatment modalities could be considered to either increase the number of targeted cells or target other precursor cells which would have a shorter lifetime than LT-HSCs. For example, in case of SCD early erythroid progenitor cells could be corrected but this would require repeated “treatment updates”, because such cells have a limited life span. In case of leukemias this may be an avenue to keep the disease under control by limiting the number of leukemic cells. However, in such examples the cost per treatment would have to come down considerably from current estimates for genetic therapy which typically exceed the million-dollar mark per patient (Leonard et al., 2022). Despite these challenges, nanomedicine holds great promise for the treatment of hematological disorders. Recent publications have demonstrated that specific targeting can be achieved *in vivo* (Wei et al., 2020; Gillmore et al., 2021; Musunuru et al., 2021; Rothgangl et al., 2021; Mullard, 2022). Intense *in vivo* screens will be necessary to determine the most optimal NP platform and modification strategy (Sago et al., 2018; Krohn-Grimberghe et al., 2020), including development of the optimal formulations for *in vivo* targeting of LT-HSCs.
